# Urea-Functionalized Heterocycles: Structure, Hydrogen Bonding and Applications

**DOI:** 10.3390/molecules28237757

**Published:** 2023-11-24

**Authors:** Soma J. Keszei, Márk Váradi, Rita Skoda-Földes

**Affiliations:** 1 Research Group of Organic Synthesis and Catalysis, University of Pannonia, Egyetem u. 10, 8200 Veszprém, Hungary; soma.keszei@univ-rouen.fr (S.J.K.); varadimark9709@gmail.com (M.V.); 2 Université de Rouen Normandie, 1 Rue Tesniere, 76821 Mont-Saint-Aignan Cedex, France

**Keywords:** ureido-heterocycles, H-bonding, self-assembly, complexation, supramolecular polymers, anion sensing

## Abstract

Ureido-heterocycles exhibiting different triple- and quadruple H-bonding patterns are useful building blocks in the construction of supramolecular polymers, self-healing materials, stimuli-responsive devices, catalysts and sensors. The heterocyclic group may provide hydrogen bond donor/acceptor sites to supplement those in the urea core, and they can also bind metals and can be modified by pH, redox reactions or irradiation. In the present review, the main structural features of these derivatives are discussed, including the effect of tautomerization and conformational isomerism on self-assembly and complex formation. Some examples of their use as building blocks in different molecular architectures and supramolecular polymers, with special emphasis on biomedical applications, are presented. The role of the heterocyclic functionality in catalytic and sensory applications is also outlined.

## 1. Introduction

Hydrogen bonds formed between neutral molecules play an excessively important role in the structure of different chemical and biological systems. The most well-known example of this phenomenon is the structure of bio-molecules’ DNA and RNA. Inspired by nature, several organic compounds with different hydrogen-bonding motifs have been developed to construct hydrogen-bonded assemblies.

Hydrogen bonds are considered weak interactions, typically with binding energy between 5 and 30 kJ/mol, which means that the introduction of multiple hydrogen-bonding functionalities is necessary to fabricate stable non-covalent molecular assemblies. However, it has long been known that the number of hydrogen bonds is not the only significant factor, but the arrangement of the hydrogen acceptor and donor groups, as well as the secondary interactions acting between them, should also be taken into consideration [1,2,3,4,5,6].

The fact that urea and thiourea derivatives are capable of forming multiple hydrogen bonds has inspired several scientists to use them in supramolecular chemistry. The first examples of urea derivatives as binding motifs were reported at the end of the last century [7,8,9,10].

In order to achieve a deeper understanding of the self-assembly properties of urea groups and to increase the strength of interactions, hydrogen-acceptor/-donor groups were attached to the urea backbone and in this regard, the best results could be obtained by using heterocyclic side chains.

**Figure 1:**
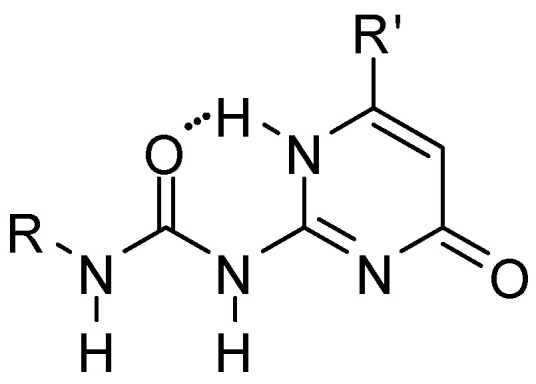
General structure of ureido-pyrimidinone (UPy) derivatives.

From the systems developed so far, the ureido-pyrimidinone (UPy) derivatives (Figure 1), introduced by Meijer [11], turned out to be the most successful. The members of this group are among the best studied synthetic multiple hydrogen-bonding systems. The reason for this is the relatively cheap and simple synthetic methodology and the ability to form four strong hydrogen bonds (*K*_assoc_ > 10^6^) in non-competitive solvents, such as chloroform or methylene chloride. This feature can be useful during the design of new hydrogen bonding polymers, molecular receptors, and in studying the competition between inter- and intramolecular hydrogen bonding. Reviews of quadruple hydrogen bonding motifs, focusing not only on ureido-pyrimidinone derivatives, but taking most of the examples from that group, appeared in 2003 [12], 2013 [13] and in 2019 (in Chinese) [14].

With the use of molecules with two or more H-bonding arrays, supramolecular structures with different architectures and tunable properties can be constructed. This area seems to be the most rapidly growing field of applications. Accordingly, supramolecular polymers based on quadruple H-bonds have been reviewed recently [15,16]. Also, a great number of reviews, summarizing the development, properties and applications of supramolecular polymers formed via different types of secondary interactions, including H-bonded arrays, have been reported. For reviews from the past three years, see refs. [17,18,19,20,21,22].

At the same time, ureido-heterocycles with triple H-bonding arrays or offering bonding patterns different from UPys can also be useful in molecular recognition, as no competition with homodimerization should be reckoned with. Also, heterocycles attached to the urea functionalities have other advantages, beyond providing further H-bond donor/acceptor sites. They are capable of binding metal ions and this can be exploited in catalysis and in the development of sensors. They can be modulated by protonation, oxidation/reduction or irradiation, which might lead to stimuli-responsive materials. To the best of our knowledge, the properties and applications of these H-bonding systems have not been reviewed before. We felt that, for the better understanding of the differences between UPys and other ureido-heterocycles, and to demonstrate the factors determining binding strength of compounds with different H-bonding motifs, properties of UPy derivatives (even if summarized in ref [13]), should also be included here. Accordingly, self-assembly and host–guest interactions of ureido-pyrimidinones, other ureido-pyrimidine, and urea derivatives incorporating other heterocycles and bearing functionalities capable of forming multiple H-bonds and developed in the past 20 years, are discussed in detail. Special emphasis will be placed on derivatives with reversibly switchable H-bonding properties that make it possible to construct stimuli-responsive material. In addition to comparing the binding properties of UPYs and other ureido-heterocycles in the formation of homo- and heterodimers, the present review is intended to give an overview of their possible applications in supramolecular chemistry, electro- and photochemistry, biomedicine, catalysis and anion-sensing, to show the great versatility of these derivatives. Because of the growing number of publications in these areas, the examples are taken from the past 10 years. Also, due to the several reviews available on the use of supramolecular polymers [15,16,17,18,19,20,21,22], just some examples will be presented to highlight the main areas of applications, without intending to be comprehensive.

## 2. Hydrogen Bonding, Self-Assembly and Complexation with Neutral Guests

Arrays of multiple hydrogen bonds are useful building blocks for the assembly of complex molecular architectures, especially owing to their directionality and specificity. Similarly to biological systems, cooperative action of certain H-bonding acceptor and H-bonding donor patterns can lead to highly specific molecular recognition events.

The magnitude of *K*_assoc_ that shows the ability for complex formation is determined by several factors, for example, by the number of the binding sites, the arrangement of the donor and acceptor groups and their secondary interactions. However, the presence of any internal hydrogen bonds, different tautomeric forms and conformational changes of the monomers, as well as solvation, can also significantly influence the stability of the assemblies.

In the design of new supramolecular arrays, it has been suggested that *K*_assoc_ (in the case of homodimers also referred to as *K*_dim_), should be comparable or greater than 10^5^ M^−1^ [23,24,25], but there are several other applications of hydrogen-bonded complexes that do not require to achieve such high tendency for association.

In the case of self-complementary quadruple hydrogen-bonding arrays, either self-assembly or, in the presence of another molecule with complementary hydrogen-bonding motifs, the formation of heterodimers is possible in non-competitive solvents. Heterodimers with good stability can also be obtained from molecules bearing non-self-complementary quadruple or triple hydrogen-bonding donor/acceptor patterns.

### 2.1. Formation of Homodimers

**Figure 2:**
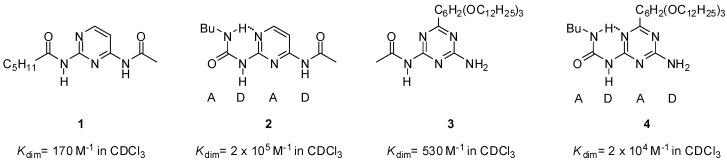
Comparison of dimerization constants for amido- (**1**, **3**) and ureido derivatives (**2**, **4**) of pyrimidine and triazine [26].

The first self-complementary quadruple hydrogen-bonding motifs (DADA) designed by Meijer and co-workers in the late 1990s were based on the urea core [26]. The higher stability of dimers of ureido compounds (Figure 2) compared to amido derivatives was attributed to a combination of favorable entropic and enthalpic effects of the intramolecular hydrogen bonds between the NH of the ureido substituent and the nitrogen atom of the heterocycle (pyrimidine or triazine).

**Scheme 1:**
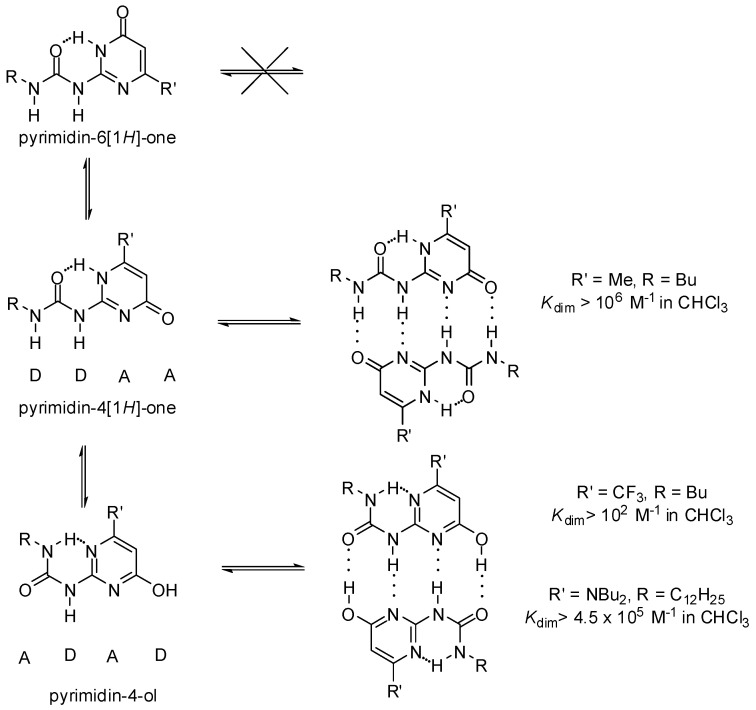
Tautomeric forms of quadruple hydrogen bonding motifs of ureido-pyrimidinones.

At the same time, due to stabilization/destabilization effects of the electrostatic attraction/repulsion between the atoms in adjacent H-bonds, the DDAA array was expected to be more stable than the DADA motif. This led to the construction of a series of ureido-pyrimidinones (Scheme 1) [11,27].

**Figure 3:**
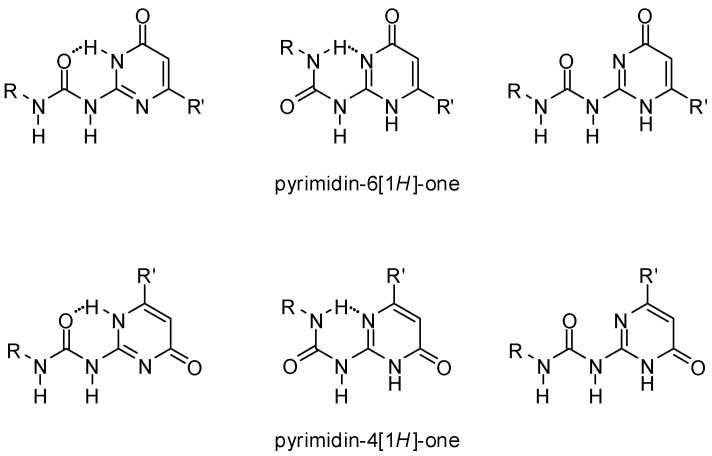
Conformers of the pyrimidin-6[1*H*]-one and pyrimidin-4[1*H*]-one tautomers of ureido-pyrimidinones.

UPys may exist in different tautomeric forms, namely the non-dimerizing pyrimidin-6[1*H*]-one tautomer, as well as two other isomers that may lead to the formation of two types of self-complementary hydrogen-bonding units with DDAA and DADA (ADAD) arrays [28] (Scheme 1). In addition, by rotation within the urea unit or around the aryl–urea bond, each tautomer may exist in several rotameric forms, some of which are stabilized by intramolecular H-bonds (Figure 3). Although undesirable tautomers are usually converted to the proper form by complexation, the phenomenon lowers association constants.

The ratio of dimers formed from the pyrimidin-4[1*H*]-one and the pyrimidin-4-ol tautomers depends strongly on the substituents, e.g., those attached to position C6 of the pyrimidine ring. Dominance of pyrimidine-4-ol type dimers was proved for compounds with either strongly electron withdrawing [11] or electron donating substituents (Scheme 1) [29,30], but their association constants were noticeably different.

**Scheme 2:**
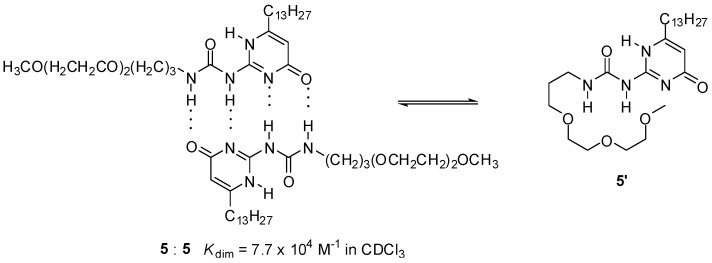
Competitive intramolecular hydrogen bonding of the side chain in an ureido-pyrimidinone (**5**) with oligo(ethylene oxide) moiety.

The presence of polar groups can also influence association strength considerably. Lower values of *K*_dim_ for UPys with oligo(ethylene oxide) side chains of a certain size (e.g., **5**, Scheme 2), compared to those with aliphatic groups, were attributed to a competitive intramolecular hydrogen bonding of an oxygen atom of the side chain, resulting in a backfolding of the chain to the polar groups of the 2-ureido-pyrimidinone moiety [31].

**Figure 4:**
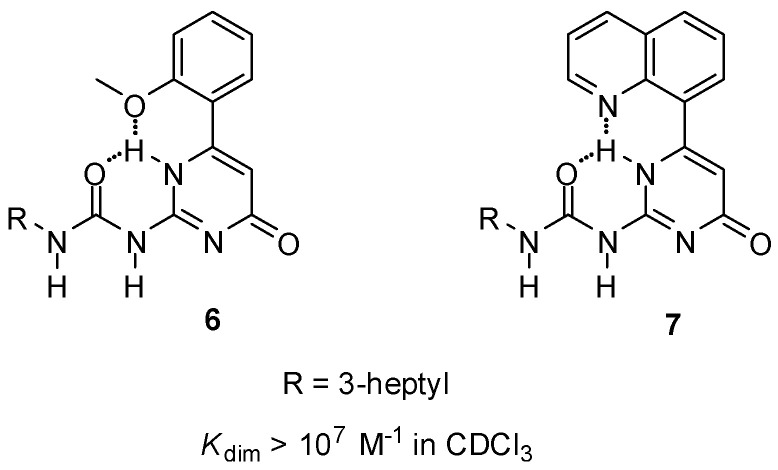
Ureido-pyrimidinones (**6**, **7**) locked in the pyrimidin-4[1*H*]-one tautomer by bifurcated intramolecular H-bonds.

Although in some cases *K*_assoc_ values were high enough for the formation of supramolecular systems [27], different strategies were adopted to avoid the change in the H-bonding patterns. By the introduction of an H-acceptor group into an appropriate position of the C6 substituent of the UPy core (**6**, **7**, Figure 4), the pyrimidine NH could be ‘locked’ by bifurcated H-bonding, which hindered tautomer formation [32]. This led to the formation of dimers with exceptionally high association constants.

**Scheme 3:**
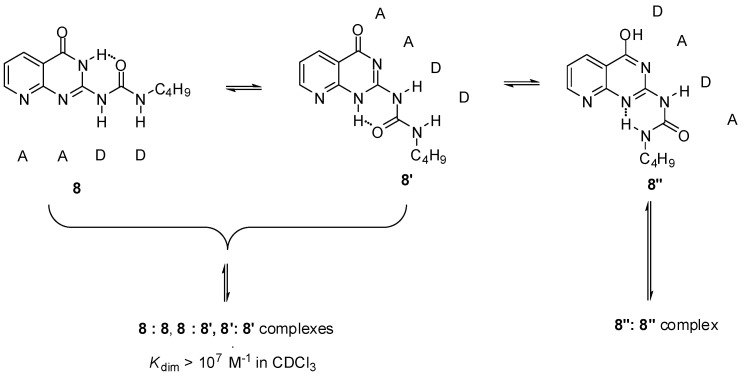
Tautomeric forms of the deazapterin derivative (**8**) developed by Zimmerman [33].

**Scheme 4:**
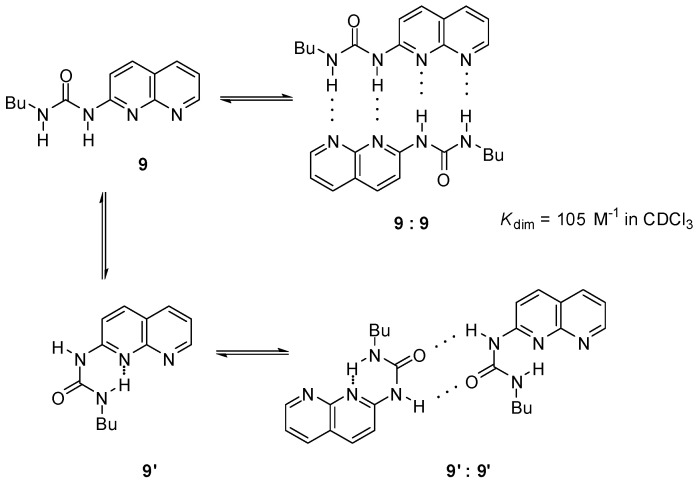
Dimer formation of conformers of ureidona-phthyridine **9**.

Another option was the replacement of the pyrimidinone core by other heterocycles. Zimmerman and co-workers reported a deazapterin derivative with an association constant exceeding 10^7^ M^−1^ bearing a DDAA binding motif (**8**, **8′**) in the two main protomeric forms. The ratio of the dimer formed from the enol tautomer (**8″**) was present in less than 2% (Scheme 3) [33]. To avoid tautomerization, 1,8-naphthyridine was attached to the urea nitrogen, instead of the pyrimidinone moiety [34] (**9**, Scheme 4). The measured association constant was much lower than was expected, which was attributed to the favorable effect of intramolecular H-bonds on the stability of dimers of **8**–**8″** (Scheme 3). As another problem, the conformational change leading to the folded form **9′** results in a dimer where the monomers are held together by only two H-bonds [35].

**Figure 5:**
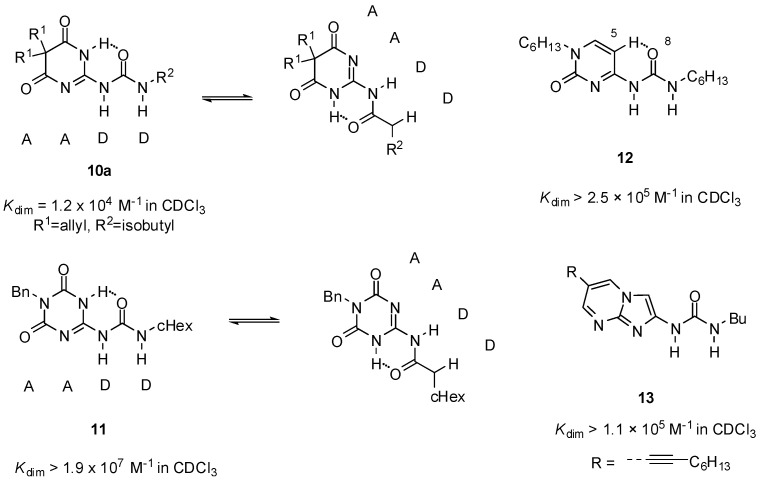
Urea derivatives with DDAA motifs.

Sanjayan introduced urea derivatives, free from tautomerization problems, by the use of degenerate prototropy. 5,5-Disubstituted-pyrimidine-4,6[1*H*]-diones (e.g., **10a**, Figure 5) [36] and 1,3,5-triazine-2,4[1*H*,3*H*]-diones (e.g., **11**) [37] show uniform DDAA arrays in either tautomeric (protomeric) forms and lead to a single dimeric structure both in solution and in the solid state. Another approach was the use of cytosine instead of pyrimidinone as the heterocyclic unit (**12**). From these molecules, stable unfolded homodimers were obtained in CDCl_3_ (*K*_assoc_ > 2.5 × 10^5^), although small amounts (5%) of folded structures held together by two H-bonds were also present in the solution [38]. By the investigation of a series of N1, C5 and N9-substituted derivatives, it was revealed that the unfolded rotamer was stabilized by the interaction of 5-H and 8-O and its ratio is increased by the introduction of longer alkyl chains on N1 [39].

No such competition between folded and unfolded conformers was observed in the case of urea derivatives with an imidazo-pyrimidine core (**13**), introduced by Hisamatsu. The existence of stable unfolded dimers with DDAA patterns was observed without any conformational competition and undesired complexes [40].

**Figure 6:**
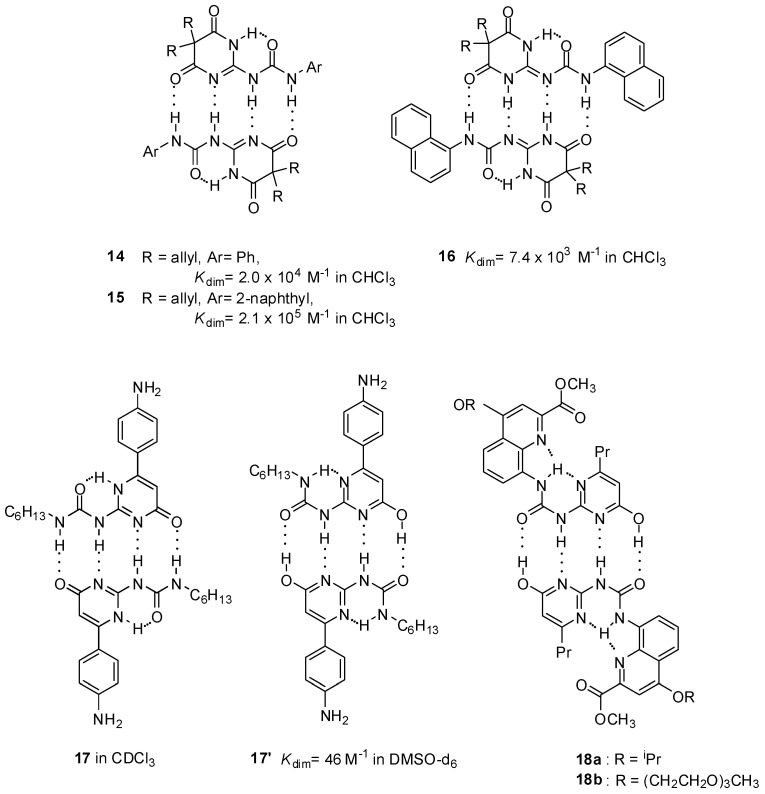
Substituent and solvent effects in dimerization via DDAA and DADA patterns.

Although the DDAA pattern was shown to be the favorable motif in obtaining stable homodimers in most cases, there are also some examples when complexation takes place via the formation of a DADA array. Interestingly, in contrast to other pyrimidine-4,6-diones (**10**, **14**, **15**), the 1-naphthyl derivative **16** was shown to form a dimeric structure of the DADA tautomers [41]. This assumption was supported in solution as well as in solid state by NMR measurements and X-ray crystallography, respectively. Dimeric structure of 6-(4-aminophenyl) derivatives of UPys were found to be dependent on the polarity of the solvent. In CDCl_3,_ they behaved in the normal manner, adopting the pyrimidin-4[1*H*]-one structure (**17**, Figure 6), though dimerization constants could not be determined due to the low solubility. On the contrary, they dimerized via the pyrimidinol form (**17′**) in DMSO, producing a complex with the DADA array [42]. Moreover, this was the first example of when a dimer with quadruple H-bonding was detected in such a polar solvent.

As another example, UPy derivatives **18a,b** were locked in the enol tautomer via the introduction of substituents, facilitating the formation of bifurcated H-bonds to generate a DADA-ADAD dimeric array. These compounds were used to construct supramolecular nanostructure morphologies in both chloroform and water, but no association constants were provided [43].

### 2.2. Formation of Heterodimers

Although quadruple hydrogen bonding patterns are the focus of investigation in most cases, examples of high affinity triple-bond systems were also reported to connect two different monomers. In these cases, only weak self-association can be expected, so the competition between self-association and hetero-complex formation usually exerts negligible effect.

**Figure 7:**
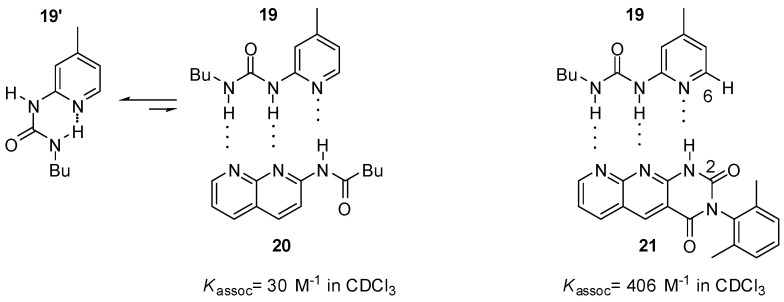
Heterodimers obtained from pyridyl-urea derivatives **19**.

Zimmerman studied pyridyl-urea derivatives with DDA binding motifs (**19**, Figure 7) [5,44]. The association constant of the complex held together by three hydrogen bonds (**19**:**20**) was rather small, as was expected. Also, complexation can take place by sacrificing the intramolecular H-bond that keeps the urea derivative in the folded conformation (**19′**). Interestingly, the heterodimer formed from pyridyl-urea **19** and tetra-azaanthracenedione (**21**) was more stable than a heterodimer held together by four hydrogen bonds with DDAD-AADA complementary patterns [44]. This could only be explained by a secondary (C-H…O) interaction between 6-H in **19** and 2-O in **21,** as well as some geometric effects [5].

**Figure 8:**
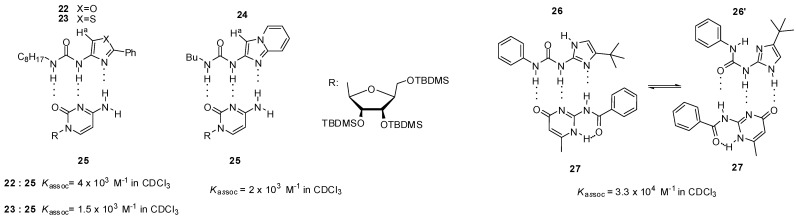
Complexation of urea derivatives of 5-membered heterocycles (**22**–**24**, **26**) with cytosine derivatives **25**, **27**.

In contrast to **19**, urea derivatives of five-membered heterocycles, such as oxazole (**22**, Figure 8), thiazole (**23**) and imidazo[1,2-a]pyridine (**24**), were shown to form more stable heterodimers with a cytosine derivative (**25**) [45]. Higher values of association constants were explained by the lack of steric hindrance between the aromatic proton (H^a^) and urea oxygen, which pushes the equilibrium towards the folded conformer that is not suitable for dimer formation in the case of six-membered heterocycles.

Wilson and co-workers investigated urea derivatives with an imidazolyl group. Although compound **26** can adopt two different conformations stabilized by internal H-bonds, both of them show the same ADD binding pattern and can form heterodimers with amido-isocytosines (e.g., **27**). This leads to a nearly three order of magnitude stronger complexation than that of the analogous pyridine derivative. (e.g., **19**, Figure 7) [41,46]. With the use of ditopic ureido-imidazole and amido-isocytosine motifs, heterodimers held together by six hydrogen bonds were also constructed [47].

**Figure 9:**
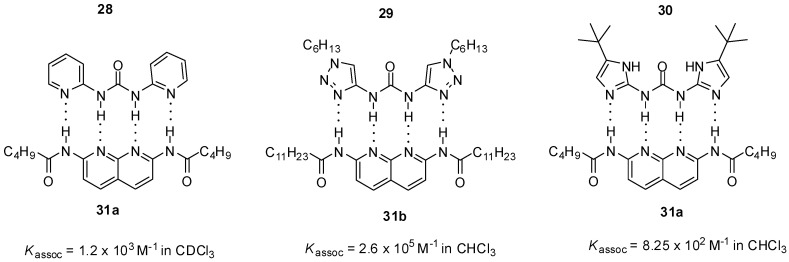
ADDA-DAAD heterocomplexes of *N*,*N*’-bis-heterocyclic derivatives of urea.

*N*,*N*’-bis-heterocyclic derivatives of urea can form heterodimers with diamido-naphthyridines through ADDA-DAAD binding motifs. Association constants were shown to depend strongly on the nature of heterocycles. The triazolyl derivative **29** formed a much more stable complex [48] than the pyridine analog **28** (Figure 9) [34,48], similarly to the difference in the behavior of the compounds with one heterocyclic ring **19** (Figure 7) and **26** (Figure 8). At the same time, unexpectedly, a weaker interaction was observed for ureido-diimidazole **30** [49], possibly due to several factors, such as energetically favored formation of a folded conformer, differences in shape complementarity between the two monomers and higher potential for self-association of **30**.

**Figure 10:**
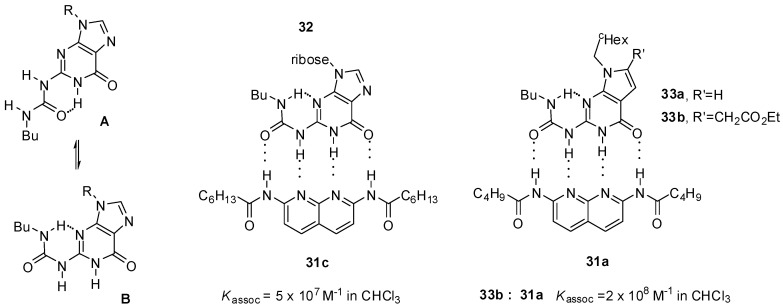
Complexation of ureido-guanine derivatives **32** and **33**. (**A**: folded conformer, **B**: unfolded onformer of ureido-guanosines).

An exceptionally high association constant was measured for a ureido-guanosine-diamido-naphthyridine complex (**32**:**31c**, Figure 10) held together by four hydrogen bonds with ADDA-DAAD pattern [50]. The guanosine derivative showed only weak self-association (with *K*_dim_ around 230 M^−1^), in contrast to deazapterin (**8**, Scheme 3) and ureido-pyrimidine (Scheme 1) derivatives. According to NMR measurements, ureido-guanosine undergoes a conformational change from the more stable unfolded conformer (**A**) to the folded form (**B**) prior to complexation.

In contrast, conformational equilibria of deaza derivatives (**33a,b**) were shown to be solvent-dependent: in DMSO-d_6_ the unfolded conformer was dominant, while in CDCl_3_ the folded form was favored. This means that these urea derivatives are preorganized in the latter solvent for complex formation, which results in association constants of approximately one order of magnitude higher than guanosine **32** during complexation with diamido-naphthyridines (**31a,c**) [51,52]. At the same time, their dimerization ability also increases (*K*_dim_ = 880 M^−1^ (for **33a**) and *K*_dim_ ≈ 600 M^−1^ (for **33b**)).

**Figure 11:**
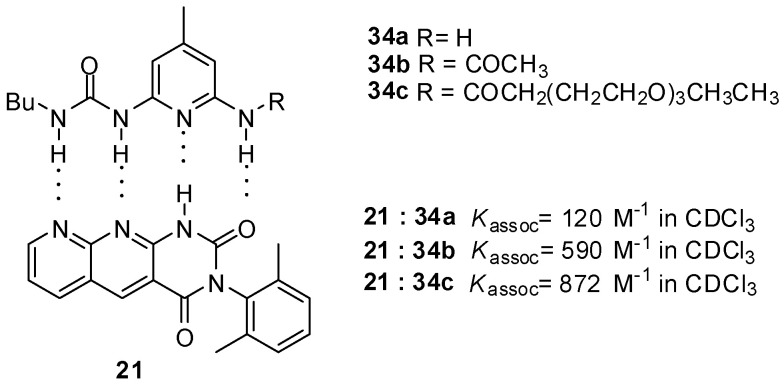
Complexation of pyridyl urea derivatives with DDAD bonding pattern (**34a**–**c**).

Similarly to the formation of homodimers, the value of binding constants was influenced considerably by the change of the substituents. Stronger binding of monomers with DDAD/AADA patterns was observed for acylated derivatives **34b,c** (Figure 11) than for the amino compound **34a** due the more acidic NH of the amides [53]. At the same time, these values show still weaker binding compared to that between quadruply H-bonded complexes with ADDA-DAAD patterns. Although the number of positive and negative secondary interactions is the same in the former and in the AADA·DDAD pair, the difference might be explained by geometric effects, i.e., a weaker association due to an inward curvature of the AADA counterpart **21** [5].

**Figure 12:**
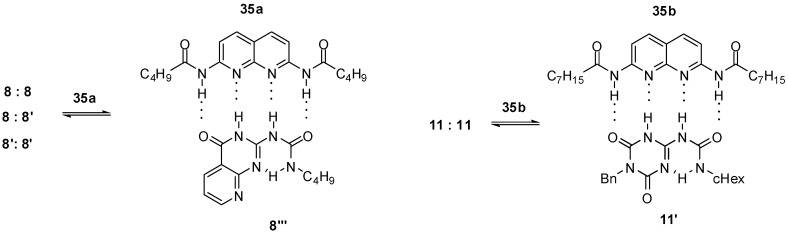
Heterodimer formation from deazapterin **8** and triazine **11**.

In the case of urea derivatives capable of forming strong homodimers via self-complementary hydrogen bonding motifs, heterodimerization should compete with self-association. In many cases, conformational changes or formation of other protomeric forms precede heterodimerization. Upon addition of diamido-naphthyridine **35a** to the urea derivative depicted in Scheme 3, a full dissociation of dimers **8**:**8**, **8**:**8′**, **8′**:**8′** and complex formation with **35a** was observed (Figure 12) [33], which supports a strong association of the two counterparts in the latter. Before heterodimerization, deazapterin **8** underwent a conformational change to exhibit the ADDA binding pattern (**8‴**). As another example, heterodimer **11′**:**35b** was formed with approximately 60% yield after addition of one equivalent of **35b** to the CDCl_3_ solution of homodimer **11**:**11** via disruption of the homodimer and a conformational change to **11′** [37].

**Scheme 5:**
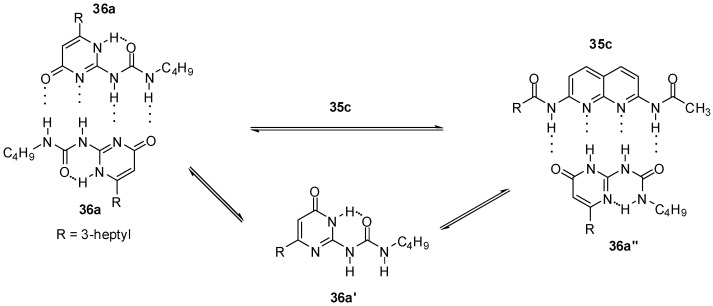
Heterodimer formation from ureido-pyrimidinone **36a**.

In the case of UPys, complex formation with 2,7-diamido-1,8-naphthyridine (e.g., **35c**, Scheme 5) takes place via the non-self-dimerizing pyrimidin-6[1*H*]-one tautomer (**36a′**). The mixture of keto- and enol type dimers (in a ratio of 90/10, for the structures of similar homodimers see also Scheme 1) was turned into an equilibrium mixture of **36a**:**36a** and **35c**:**36a″** dimers, while the enol homodimer was found to disappear very quickly [30].

**Figure 13:**
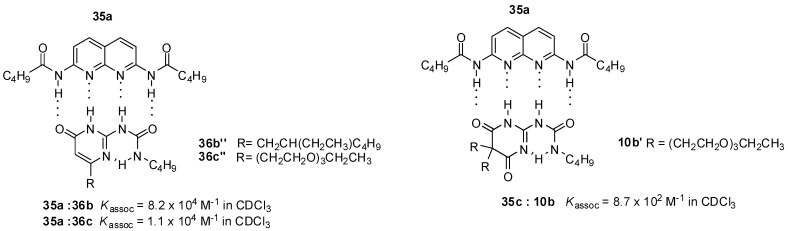
Heterodimer formation from ureidopyrimidinones **36b,c** and ureido-pyrimidinedione **10b**.

Strong association between the 3-cyano-diamido-naphthyridine (**35d**) derivative and UPys bearing solubility-enhancing substituents (**36b,c**) was observed by Lüning, based on NMR measurements (Figure 13) [54]. In contrast, the folded conformer (**10b′**) of pyrimidinedione (**10b**), constructed to avoid the problem of formation of different tautomeric forms, developed much weaker interaction with the same guest. This was assumed to be the result of the sp^3^ carbon atom in the heterocycle that led to a distortion of the ring and so allowed an easier contact between the polyethylene glycol chain and the binding site and hindered association.

**Scheme 6:**
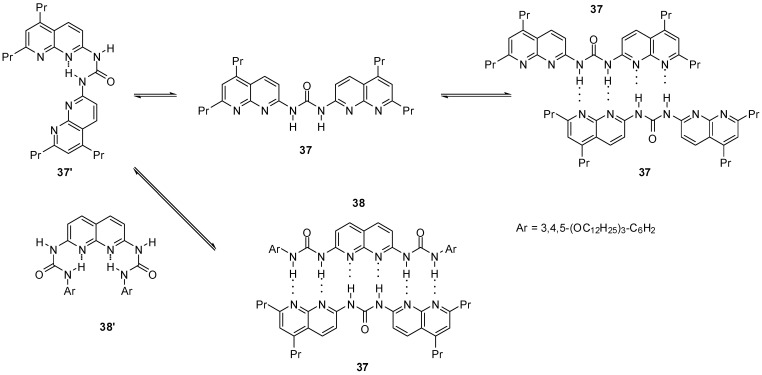
Heterodimer of bis-naphthyridine **37** and bis-urea **38**.

Bis-naphthyridine **37** (Scheme 6) and bis-urea **38** were shown to exist in the folded conformations (**37′** and **38′**) in solution. At higher concentrations, the latter was found to self-dimerize via the formation of the unfolded conformer (**37**). In the mixtures of the two components a robust, sextuply H-bonded complex (**37**:**38**) was obtained by the association of the unfolded conformers of each monomers [55].

**Figure 14:**
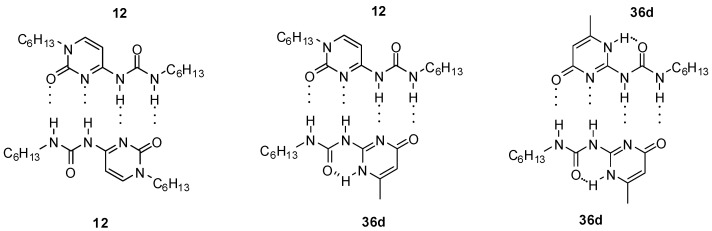
Homo- and heterodimers formed from ureido-cytosine **12** and ureido-pyrimidinone **36d**.

Heterodimers (**12**:**36d**, Figure 14) were present in a considerable amount in the 1:1 mixture of self-associating ureido-cytosine (**12**, *K*_dim_ > 9 × 10^6^ M^−1^ in C_6_D_6_) and ureido-pyrimidinone (**36d**) derivatives (ratio 5:6:5), which shows the strength of interaction between the two different monomers [38].

**Scheme 7:**
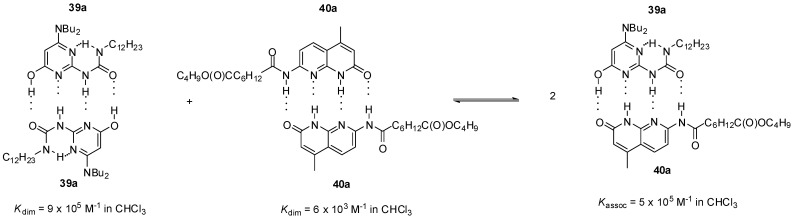
Heterodimer formation from homodimers of amino-ureido-pyrimidinone **39a** and amido-naphthyridone **40a**.

As another example, heterodimer formation was also detected between the pyrimidin-4-ol tautomer of amino-ureido-pyrimidinone **39a** and amido-naphthyridone **40a** (Scheme 7) [56]. According to calculations, the free energy change for the formation of heterocomplexes from homodimers is negative, so the heterocomplex should be favored over the latter in equimolar mixtures of the components. NMR studies revealed that, indeed, the ratio of heterocomplex **39a**:**40a** was 73% at 263 K.

In order to mimic signaling pathways of biological systems, sequential self-sorting cascades were developed with the use of multiple components capable of self-association and/or heterodimer formation.

**Scheme 8:**
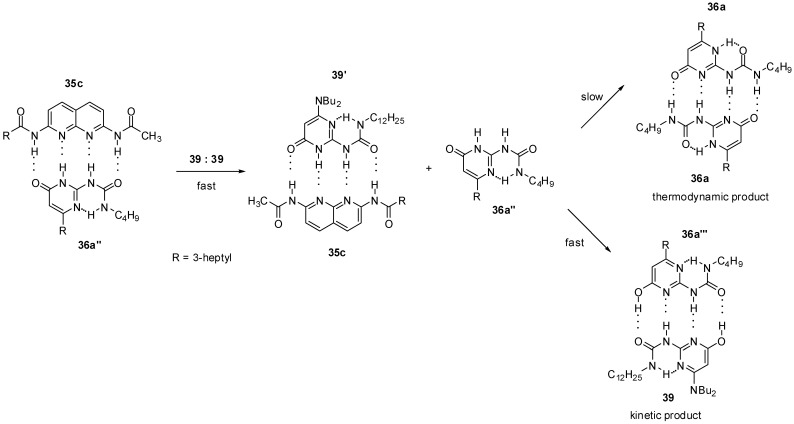
Formation of the thermodynamically disfavored heterodimer **36a‴**:**39**.

The thermodynamically disfavored heterodimer (**36a‴**:**39**, Scheme 8) could be obtained as a kinetic product by mixing the 2,7-diamido-1,8-naphthyridine (**35c**): ureido-pyrimidinone (**36a″**) heterodimer with the homodimer of the 6-amino-ureidopyrimidinone derivative **39** [30]. In addition, **36a** was shown to be released from dimer **35c**:**36a″** in the pyrimidin-6[1*H*]-one form (**36a″**) that could rapidly turn into its tautomeric enol form (**36a‴**) via an [1,3]-shift and is associated with **39**. The formation of the pyrimidin-4[1*H*]-one (**36a**) homodimer would require both a [1,3] proto-tropic shift and the break of an intramolecular hydrogen bond, so it is a much slower process.

**Figure 15:**
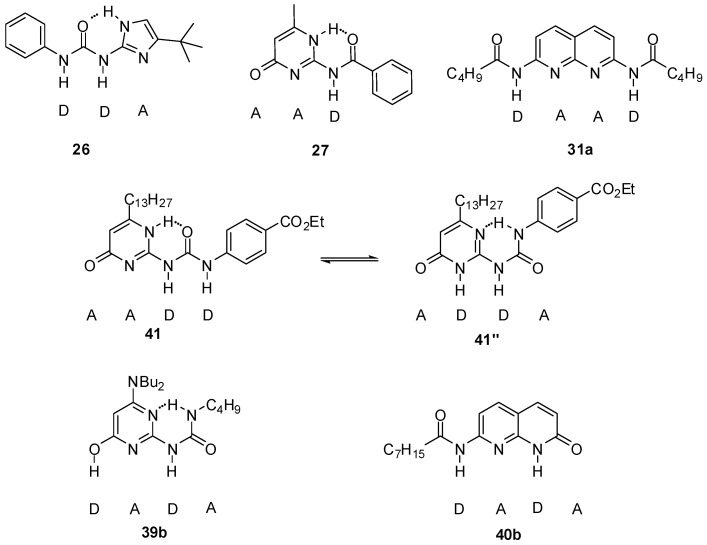
Components of molecular cascades built by Wilson et al. [57,58].

A four-component molecular cascade was built by Wilson et al. [57]. The formation of two heterodimers (**26**:**27** and **31a**:**41″**) as the main products was observed in the equimolar mixture of ureido-imidazole **26**, amido-cytosine **27**, a bis-amido-naphthyridine (**31a**) and ureido-pyrimidinone **41** (Figure 15). The two heterodimers could also be obtained upon sequential addition of **26** → **27** → **31a** to the UPy homodimer **41**:**41**. From the results, it was concluded that product distribution was not governed solely by the value of association constants and the main driving force in determining product distribution was to maximize non-covalent interactions in the entire system. At the same time, it is advantageous if one of the components recognizes a limited number of other derivatives (in the present case, compound **27**).

This network was extended by two further derivatives, amino-ureido-pyrimidinone **39b** and amido-naphthyridone **40b** [58]. It was shown that the product distribution of the systems obtained by sequential additions of up to five of the six building blocks can be understood by detailed NMR analysis of molecular recognition behavior of hydrogen-bonding motifs, supplemented with calculations.

**Scheme 9:**
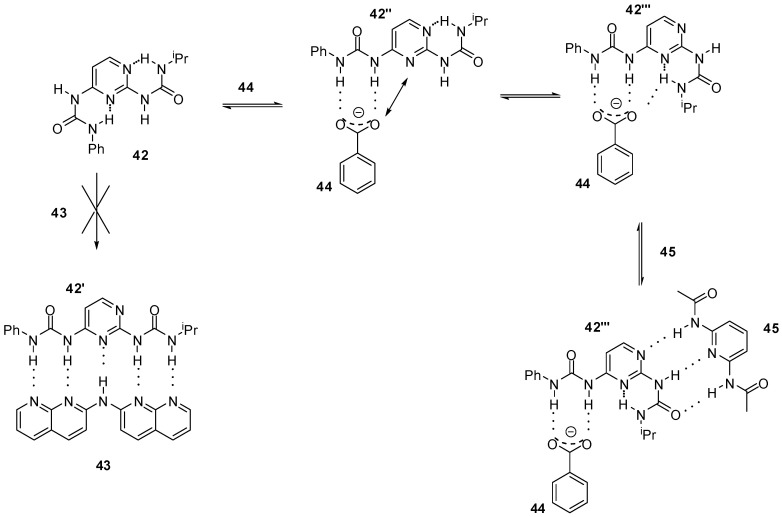
The behavior of bis-urea **42** towards anionic and neutral counterparts.

In the examples discussed above, a conformational change often precedes heterodimerization, accompanied by disruption of intramolecular H-bonding. Ośmiałowski et. al. found that even the formation of five intermolecular hydrogen bonds (e.g., with guest **43**) were not enough to change the thermodynamically preferred conformation of bis-urea **42** bearing two intramolecular H-bonds (Scheme 9) [59]. In contrast, the addition of benzoate ions (**44**) resulted in a complexation with conformer **42″**. This was found to be followed by a conformational change to **42‴** due to the electronic repulsion between benzoate oxygen and the non-substituted N3 of the pyrimidine ring in **42″**. Moreover, a bifurcated H-bond could also stabilize conformer **42‴**. The latter isomerization opened up the way for association of the bis-amido-pyridine guest **45** via an ADA/DAD bonding pattern resulting in the terner complex of **44**:**42a‴**:**45**.

### 2.3. Incorporation of Stimuli-Responsive Moieties into Urea Derivatives Functionalized by Heteroaromatic Groups

Sufficient increase in temperature evidently results in the disruption of H-bonds. This is reversible in most cases: the counterparts with the proper motifs can be reassembled upon cooling. Beside the change in temperature, H-bonding ability can be altered considerably by reduction/oxidation or photochemical reactions if redox- or photoactive moieties are introduced either into heterocyclic ureas or into the derivatives that can serve as their counterparts in complexation reactions. Also, the H-bonding motif can be modified by protonation/deprotonation [60]. Investigation of these features can lead to the development of redox-active sensors or switchable supramolecular assemblies.

#### 2.3.1. Redox-Active Urea Derivatives

By the introduction of electroactive moieties into heteroaromatic urea derivatives, complexation processes can be followed not only by NMR and UV/Vis spectroscopy, but also by electrochemical methods, e.g., by cyclic voltammetry (CV). As another feature, the strength of H-bonds can be altered by electron transfer to create redox-responsive H-bonded complexes [61,62] that can result in the development of stimuli-responsive formation of supramolecular polymers.

**Figure 16:**
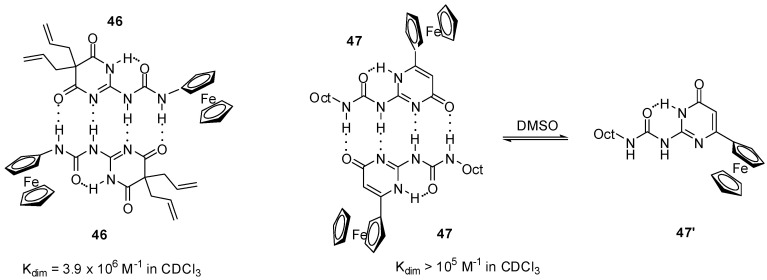
Dimeric structures obtained from ferro-cenyl compounds **46** and **47**.

Similarly to simple derivatives, ureidopyrimidin-4,6-diones (e.g., **46**, Figure 16) and -pyrimidin-4-ones (e.g., **47**) decorated with ferrocenyl moieties were found to form stable dimers in apolar solvents, such as CDCl_3_ or CD_2_Cl_2,_ and were shown to exist as monomers (in the case of **47**, in the pyrimidin-6[1*H*]-one form **47′**) in polar media, e.g., in DMSO-d_6_ and CD_3_CN, as supported by NMR measurements [63,64].

In accordance with these observations, two reversible oxidation waves with similar current levels were detected for dione **46** by CV in CH_2_Cl_2_ [63]. At the same time, increased current levels for the first oxidation wave and gradually decreasing levels for the second one at more positive potential were obtained in CH_2_Cl_2_/CH_3_CN mixtures, while only one wave could be observed in acetonitrile. It was concluded that the two equivalent ferrocene centers in the dimer exhibited a remarkable level of electronic communication as they underwent oxidation at very different half-wave potentials. In contrast to this, two waves with different levels of current were detected in CH_2_Cl_2_ for compound **47**, while one reversible wave was obtained at lower potential after the addition of DMSO (30%) [64]. The phenomenon was attributed to the pyrimidin-4-one dimer (**47**:**47**) → pyrimidin-6-one monomer (**47′**) transition.

Another group suggested that the observation of the two oxidation and two reduction peaks was due to electronic communication between the two ferrocene units [65], similarly to the pyrimidin-4,6 dione derivatives (**46**) [63]. It should be emphasized that, in contrast to the behavior of the latter derivative, there was a great difference in the current levels of the two waves in the case of compound **47**.

**Scheme 10:**
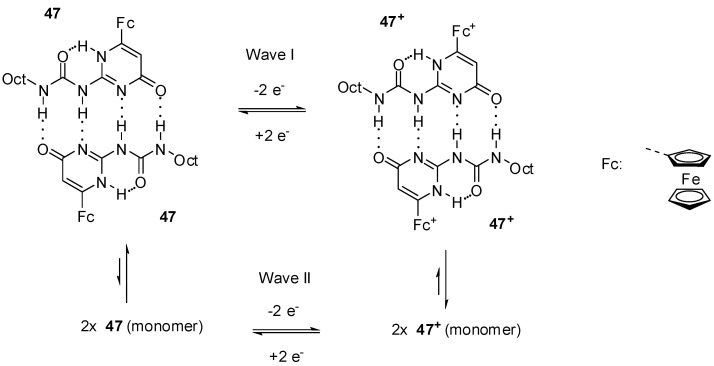
Equilibria between dimers and monomers of reduced and oxidized forms of ferro-cenyl derivative **47**.

Smith and co-workers reported a detailed study on the octyl-derivative (**47**) [66] and their results supported the assumption of Cooke’s group [64]. They suggested that the pyrimidin-4-one dimers broke up due to electrochemical oxidation in CH_2_Cl_2_. According to their results, these phenomena can be caused by a combination of electrostatic repulsion and decreased H-accepting ability of the oxidized species. They supposed that, on the CV time scale, the most probable product was the pyrimidin-4-one monomer (**47**) that tautomerized to the pyrimin-6-one monomer (**47′**) afterwards. The two waves with different currents can be attributed to the equilibrium (Scheme 10) of the dimer (major component) and monomer (minor component) in the reduced form, and dimer (minor component) and monomer (major component) after oxidation.

**Figure 17:**
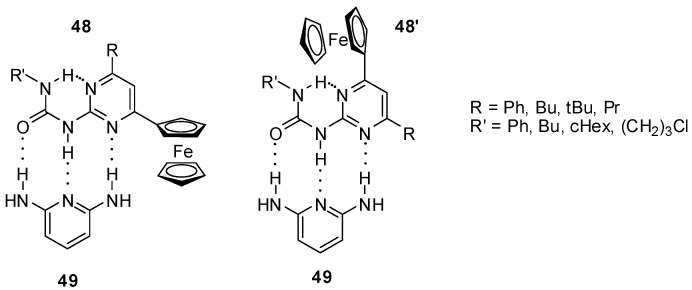
Host–guest interaction between ferrocenyl-ureido-pyrimidines (**48**) and 2,6-diaminopyridine (**49**).

NMR studies proved the existence of an equilibrium between the two folded conformers of 2-ureido-4-ferrocenylpyrimidines (**48** and **48′**) bearing an intramolecular hydrogen bond [67]. Both conformers were shown to establish host–guest interaction with appropriate guests, such as 2,6-diaminopyridine (**49**, Figure 17) with a donor–acceptor–donor H-bonding pattern. Complexation could be followed by the anodic shift of *E*_pa_ values.

**Figure 18:**
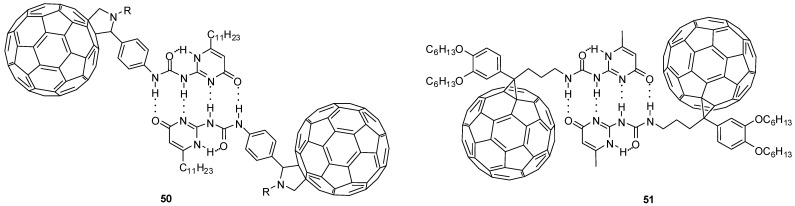
Fullerene-decorated UPy dimers (**50**, **51**) formed by multiple hydrogen bonds.

Fullerene derivatives have interesting properties that make them ideal building blocks in organic solar cells or functional materials. Several fullerene-labelled hydrogen-bonded molecular assemblies (e.g., **50** and **51**, Figure 18) were synthesized and investigated, using Meijers quadruple hydrogen-bonding ureido-pyrimidines [68,69,70]. Their electrochemical investigations revealed that the fullerene moieties are oxidized simultaneously and there is no electronic communication between the two units.

**Figure 19:**
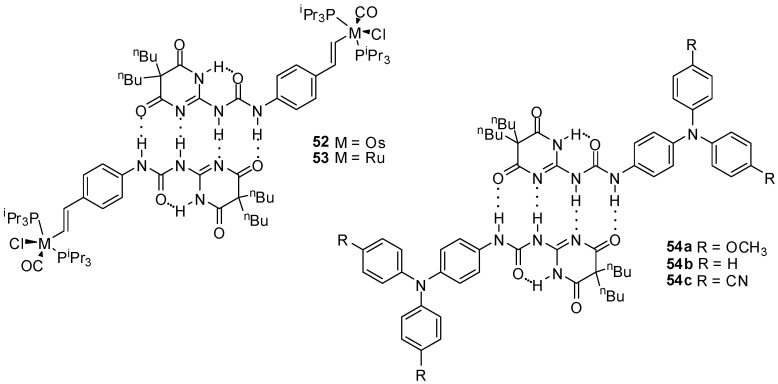
Dimeric Os and Ru complexes (**52**, **53**) and triarylamine derivatives (**54a**–**c**) held together by quadruple H-bonding.

Zális and co-workers studied Os and Ru complexes of styryl-functionalized ureidopyrimidin-4,6-dione derivatives, forming stable dimers due to quadruple hydrogen bonding (Figure 19). The existence of dimers was proved by X-ray crystallography, ^1^H NMR measurements and infrared spectroscopy, as well as by conventional electrochemical methods and UV/Vis spectro-electrochemistry. Similarly to the fullerene derivatives, no electronic communication could be detected in these complexes between the two redox centers, which was attributed to the poor contribution of the groups involved in the hydrogen bonding to the occupied frontier molecule orbitals [71].

Two well-defined waves were observed in the voltammograms of solutions of triarylamine derivatives (**54a**–**c**) in CH_2_Cl_2_ [72] that could be explained by the sequential oxidation of the two NAr_3_ units to give a one- and a two electron-oxidized species. This was the first example for a proton coupled organic mixed valency in solution, where the stability of the dimers could be controlled by varying the substituents of the triarylamine moiety.

**Figure 20:**
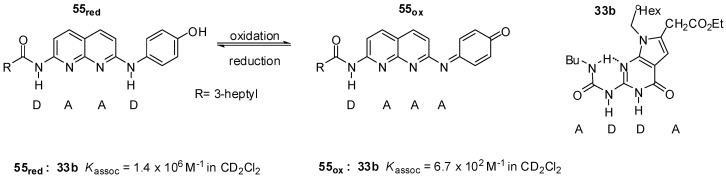
Redox switchable system of deaza-guanosine **33b** and naphthyridine **55**.

Zimmerman et. al. developed a redox switchable system composed of deaza-guanosine **33b** and the naphthyridine derivative **55** (Figure 20). In its reduced form, the latter can form heterodimers with the urea derivative **33b** held together by strong DAAD:ADDA arrays. In contrast, the oxidized compound with the DAAA pattern showed a much weaker association [73].

**Scheme 11:**
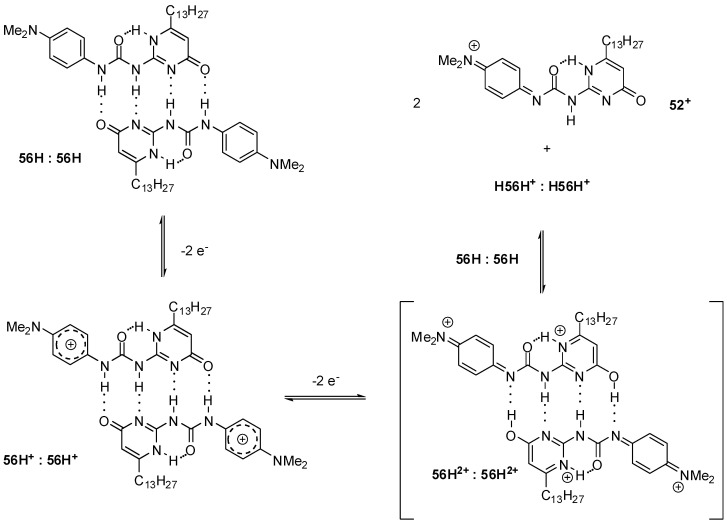
Hydrogen-bonded supramolecular systems manipulated by an electrochemically induced proton-coupled electron-transfer reaction.

Smith demonstrated an example of the splitting of the hydrogen bonds in a phenylenediamine-labelled UPy (**56H**, Scheme 11) assembly by an electrochemically induced proton-coupled electron-transfer reaction. The difference in the intensity of the two oxidation waves was explained by a mechanism in which the original DDAA bonding pattern (in the **56H**:**56H** dimer) is converted to the weaker DADA array (in **56H^2+^**:**56H^2+^**). Due to this and also to the greater electrostatic repulsion of the positively charged components, the equilibrium is shifted towards the monomers. Their highly acidic protons can protonate another dimer in the reduced form (**56H**:**56H**), which leads to quinoidal cation monomers (**56^+^**) and protonated reduced dimers (**H56H^+^**:**H56H^+^**) [74].

#### 2.3.2. Photo-Switchable Derivatives

**Scheme 12:**
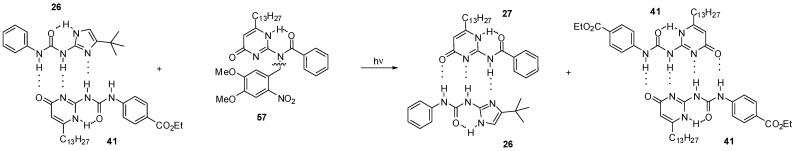
Photochemically triggered dimerization imidazole-urea **26** and amido-isocytosine **27**.

During the investigation of self-sorting cascades, Wilson et al. developed a photo-triggered dimerization between imidazole-urea **26** and amido-isocytosine **27** (Scheme 12). While the *N*-benzyl derivative **57** did not destroy either **41**:**41** or **26**:**41** dimers, the mixture of **57** and dimer **26**:**41** could be converted to a mixture of the homodimer **41**:**41** and the heterocomplex **26**:**27** via photochemical cleavage of the *N*-benzyl group [57].

**Scheme 13:**
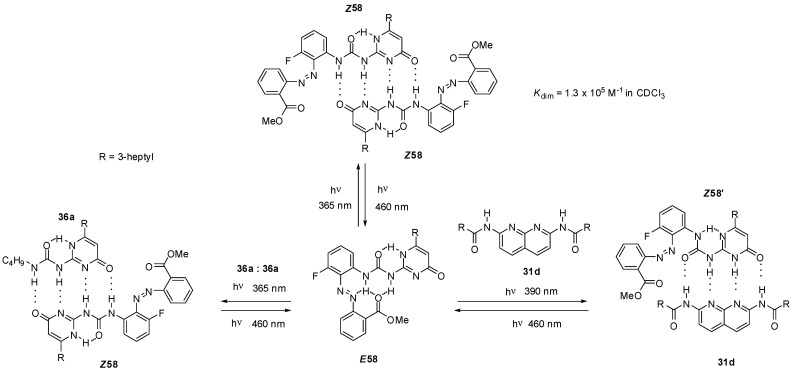
Photo-triggered homo- and heterodimerization of azobenzene ***E*58**.

**Scheme 14:**
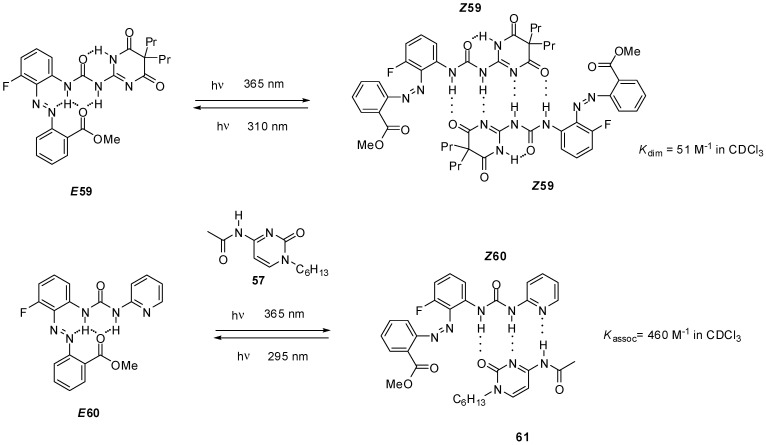
Photo-triggered dimerization of azobenzene-decorated pyrimidinedione- (***E*59**) and pyridyl derivatives (***E*60**).

Ureido-pyrimidinone with an azobenzene unit (**58**, Scheme 13) was shown to undergo dimerization only after a UV irradiation induced isomerization (from ***E*58** to ***Z*58**) that disrupted the intramolecular H-bonds and made the DDAA bonding pattern available for dimer formation [75]. The process could readily be reversed by blue light irradiation. The azobenzene derivative ***E*58** was also capable of establishing quadruple hydrogen bonds, either with non-labelled ureido-pyrimidinone **36a** [75] or bis-amido-naphtyridine **31d** [76] under similar conditions. In the latter case a conformational change was also necessary to adopt the ADDA H-bonding pattern (***Z*58′**). Homo- and heterocomplexes could be constructed reversibly from pyrimidinedione (***E*59**, Scheme 14) and pyridyl derivatives (***E*60**), respectively, although association constants were considerably lower than those observed for the azobenzene-substituted UPy ***E*58** [77]. Photoisomerization of similar systems can lead to development of photo-responsive supramolecular polymers [78].

#### 2.3.3. pH-Switchable Derivatives

**Scheme 15:**
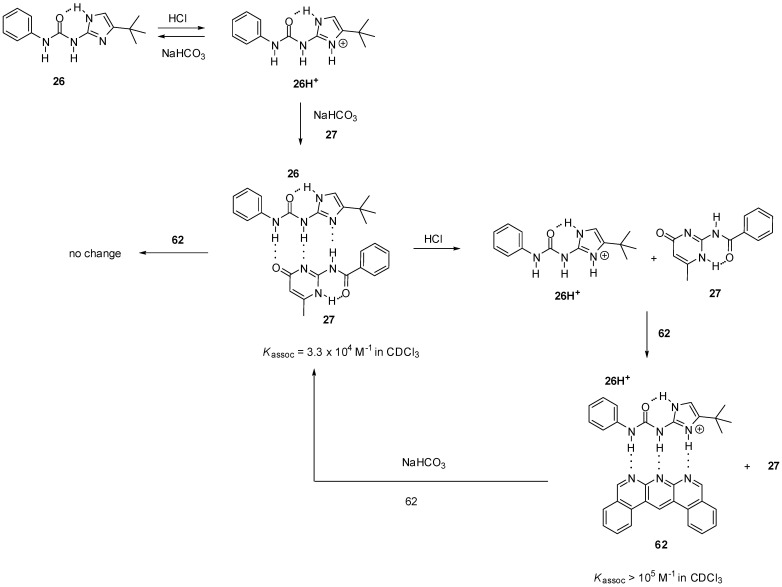
Heterodimer formation of ureido-imidazole **26**, governed by protonation/deprotonation reactions.

H-bonding patterns can also be modulated by protonation/deprotonation reactions. Wilson et al. showed that, by switching the DDA array of the imidazole derivative **26** into a DDD motif (**26H^+^**, Scheme 15), the complexation behavior of the compound could be altered [79]. While no interaction was observed between the protonated derivative **26H^+^** and amido-isocytosine **27**, the formation of the heterodimer could be proved by NMR after the addition of sodium hydrogen carbonate. No change in the ^1^H NMR spectrum could be noticed upon addition of the benzo-isoquinolino-naphthyridine **62** to the heterodimer **26**:**27**, which showed that the interaction could not be disturbed due to the much smaller association constant of the complex **26**:**62** (*K*_assoc_ = 2 × 10^3^ M^−1^ in CDCl_3_). In contrast, naphthyridine **62** could construct a heterocomplex with the protonated derivative **26H^+^** even in the presence of amido-isocytosine **27**.

## 3. Formation of Different Molecular Architectures via Supramolecular Assembly

In the first years after Meijer’s report of self-assembling UPys, great interest turned to the application of heterocyclic urea derivatives in nanotechnology and supramolecular polymer chemistry. The ability to form hydrogen-bonded assemblies [12] can be exploited in the field of self-healing gels, synthesizing either modified ureido-pyrimidine oligomer chains or by attaching ureido-pyrimidines to a polymer backbone [23]. These polymers can be broken down by heating via splitting the hydrogen bonds that can be regenerated after cooling. In case of electrochemically active ureido-pyrimidines, redox-switchable polymers can also be obtained.

**Figure 21:**
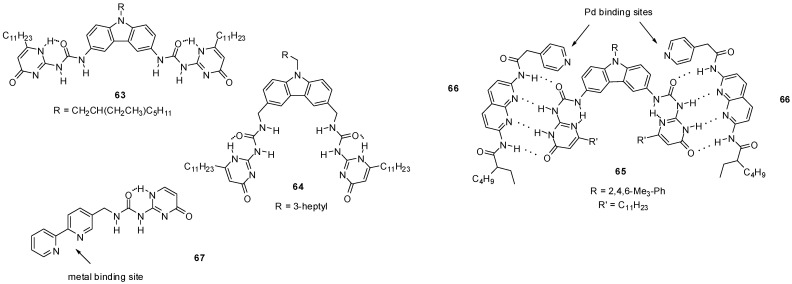
UPy derivatives of 3,6-carbazole (**63**–**65**) and 2,2′-bipyridine (**67**).

Meijer discovered that, besides supramolecular polymers, bifunctional UPys are also able to form small cyclic oligomers in CHCl_3_. The equilibria between open chain polymers and cyclic oligomers of UPy derivatives were investigated by ^1^H NMR and viscosimetry. It was found that the use of alkyl linkers with methyl substituents next to the UPy moieties changed the preferred conformation of the monomers, which resulted in a shift of the equilibrium towards the cyclic dimers in solution. This means that, by the choice of the substitution pattern and length of the linker moiety, the ratio of cyclic oligomers and long-chain polymers can be influenced [80]. The importance of the structure of linker groups was also supported by the work of Mendoza et al. The bifunctional UPy derivative **63** (Figure 21), bearing a 3,6-carbazole linker, was found to lead to a viscous polymeric structure, while the insertion of methylene spacers resulted in the formation of cyclic aggregates of **64** with preference for a tetrameric structure [81].

Cyclic three-component molecular complexes were constructed by quadruple H-bond formation between dimeric UPy units (**65**) and bis-amido-naphthyridines (**66**) decorated with ligands capable of binding metals. By the addition of neutral Pd-precursors, **65**_2_**66**_4_Pd_2_-type cyclic complexes were formed [82]. A combination of metal coordination and self-assembly of UPy motifs led to the construction of tetrahedral cages from bipyridine derivative **67** and Fe^2+^ or Zn^2+^ ions, even in polar solvents, such as in acetonitrile and methanol [83].

**Figure 22:**
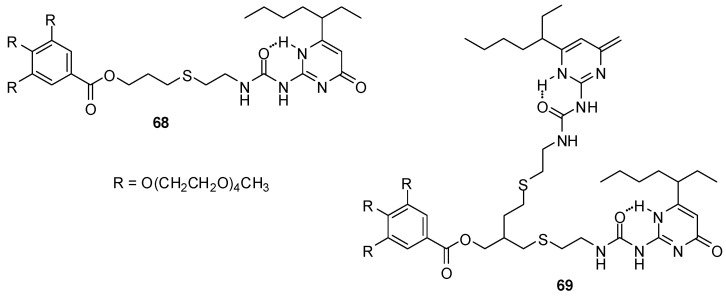
UPy derivatives forming micellar structures in water.

UPys decorated with low molecular weight tetra-ethylene glycol (TEG) monomethyl ether chains were shown to assemble into vesicles or micelles in water depending on the number of hydrogen bonding units [84]. According to TEM, cryo-TEM and dynamic light scattering measurements, compound **68** (Figure 22) dimerizes through H bonding into bola-amphiphiles that form vesicles via self-assembly. In contrast, the ureido-pyrimidine units of bis-UPy **69** were found to cross-link with each other and to form random-coiled supramolecular polymers that led to the formation of micelles. With the help of Nile Red encapsulated into these micelles, it was shown that they could be disrupted under basic conditions, possibly by the altered hydrogen bonding pattern due to the formation of enolate via the deprotonation of the enol form. This shows that these and similar molecules can find applications in drug delivery.

**Figure 23:**
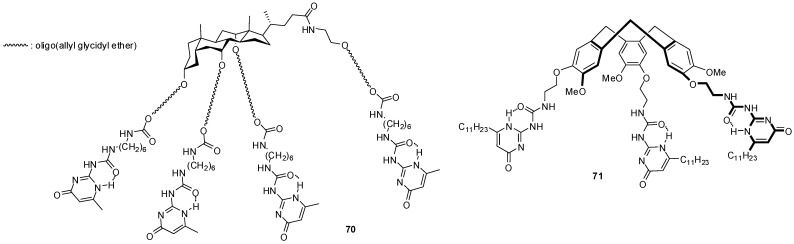
Star-shaped molecules with multiple UPy units.

The star-shaped derivatives of bile acid with UPy end groups (**70**, Figure 23) were shown to form micelles in DMSO with the nonpolar cholic acid skeletons in the micellar core and polar chains pointing towards the solvent [85,86]. Hydrogen bonding between the pyrimidinone units was thought to be suppressed by DMSO. By the addition of water, first the formation of fibrillary aggregates was observed, which turned into spheres by further aggregation. The changes are thought to be the result of an increase in the number of hydrogen bonds between the side chains and also between the micelles with UPy moieties on the surface. Although water usually inhibits dimerization, hydrophobic hexyl chains are believed to form hydrophobic pockets that facilitate the formation of hydrogen-bonded dimers.

**Figure 24:**
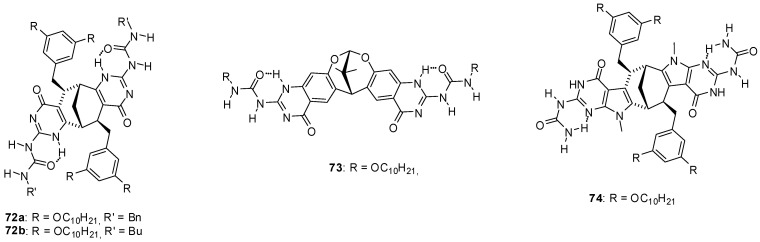
UPy derivatives with dioxabicyclo[3.3.1]nonane framework.

Reversible formation of dimeric capsules from other star shaped molecules (**71**) was used for the purification of fullerenes that could be encapsulated in the cavity [87]. Moreover, a remarkable selectivity for the inclusion of C_70_ over C_60_ was observed. Other fullerene encapsulating assemblies were built from enantiomerically pure monomers (**72a,b**, Figure 24) obtained by fusing two UPy units to the bicyclo[3.3.1]nonane framework [88]. Detailed studies revealed a series of interesting features of these structures. A mixture of cyclic pentamers and tetramers was obtained in toluene, while the tetrameric form was the only structure detected in CDCl_3_. By mixing the toluene solutions of the two very similar monomers, a scrambling could be detected after an aging period resulting in tetramers and pentamers containing both monomers. At the same time, no such change in the structures of cyclic oligomers took place in CDCl_3_. An association constant of *K*_assoc_ = 1.16 × 10^5^ M^−1^ was measured for C_60_:**72a**_4_ complexes and an even stronger interaction could be observed with C_70_ due to the larger π-surface of this fullerene. As another feature, the encapsulation that could be observed in toluene, but not in CHCl_3_, hindered scrambling between the two types of monomers **72a** and **72b**. In contrast to these compounds with the bicyclo[3.3.1]nonane framework, only the tetramer of the dioxabicyclo[3.3.1]nonane derivative **73** was observed, either in CHCl_3_ or in benzene [89].

**Figure 25:**
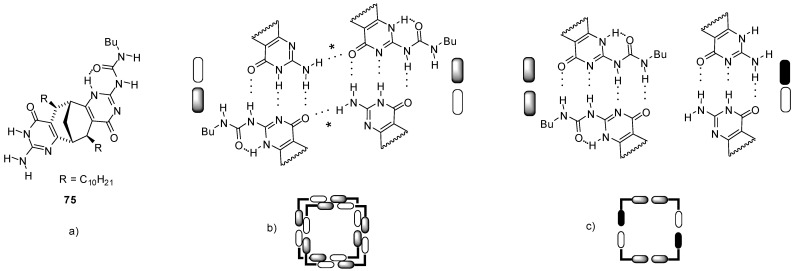
(**a**) Bicyclo[3.3.1]nonane **75** with one UPy and one ICyt functionality. (**b**) Formation of tubular octamers via H-bonding (*) of tetrameric forms. (**c**) Tetrameric form with H-bonded UPy-UPy and ICyt-ICyt’ units.

A monomer with the same bicyclo[3.3.1]nonane backbone but containing one UPy and one iso-cytosine (ICyt) motif (**75**, Figure 25a) was found to form tetrameric cyclic aggregates via three complementary H-bonding motifs (UPy-ICyt) at the junctions (Figure 25b) [90]. With the formation of further H-bonds (labelled with an *), two tetramers were shown to assemble to a tubular octamer. By the addition of C_60_ in toluene, this octamer was found to rearrange into a complex C_60_:**75**_4_ by changing the H-bonding pattern in the tetramers into UPy-UPy and ICyt-ICyt’ connections with DDAA/AADD and DDA/AAD motifs, respectively (Figure 25c).

Another switchable assembly was developed based on monomer **74** (Figure 24) that adopted the tetrameric form in CDCl_3_ [91]. At the same time, by using toluene as solvent or upon addition of a C_70_ guest, aggregation to tubular supramolecular polymers was observed.

It should be mentioned that, by the integration of orthogonal self-assembly of H-bonding motifs and macrocyclic host–guest interactions, supramolecular polymers of diverse structures and with interesting properties were obtained, as reviewed in 2020 [92].

**Figure 26:**
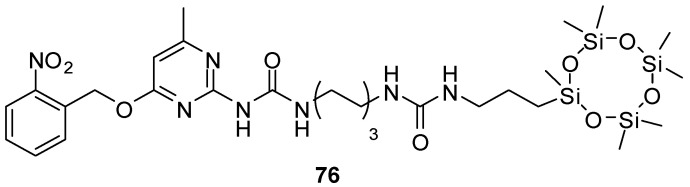
Nanosheet-forming UPy derivative **76** with a cyclo-siloxane moiety.

Various derivatives with UPy motifs were shown to assemble into two-dimensional nanomaterials that drew great attention in various applications, such as in chemical sensors. The water-soluble derivative **18b** (Figure 6) locked in the enol tautomer was shown to form nanosheets even in water. Hydrogen bonds remained undisturbed even in water due to the shielding effect of the hydrophobic π-stacking pockets of quinolone [43]. As another example, aggregation of the molecules of UPy derivative **76** (Figure 26), bearing a cyclo-siloxane moiety as the hydrophobic unit, led to the formation of large-area nanosheets with a lamellar structure [93].

## 4. Applications Exploiting the H-Bonding Ability of Ureido-Heterocycles

### 4.1. Supramoleular Polymers

The ability of multiple H-bonding motifs to connect different molecular entities with relatively strong secondary interactions that can usually be disrupted and reassembled in a reversible way makes them ideal building blocks in large molecular assemblies. The great interest shown towards these applications is demonstrated well by the several reviews that appeared recently covering a great variety of topics. As a consequence, only the main areas are summarized here with reference to these reviews and in some cases some specific examples are highlighted.

Macromolecules bearing hydrogen bonding end-groups can generally be synthesized by post-polymerization functionalization or by the use of functional precursors to introduce hydrogen bonding groups to the polymers [94]. The integration of H-bonding subunits into different positions of the monomer strand not only aids the evolution of the polymer frame, but it also helps in creating a matrix that is increased in strength and tolerates defects. This could be explained by the energy-dissipating character of the sacrificial H-bonds [95].

The capability to form multiple H-bonds makes the ureido-pyrimidine moiety an ideal unit in supramolecular polymers, i.e., in polymers in which the monomers are crosslinked physically. The non-covalent interactions can be useful in the design of elastomers, self-healing polymers, bioactive materials and molecular catalysts. Although there are some examples of supramolecular polymers of ureas constructed using triply [96] or sextuply H-bonded units [97], the most common motifs are those capable of forming quadruple H-bonds [15,98,99].

Self-healing in materials means the re-formation of disrupted chemical bonds that leads to the regain of original mechanical properties. Polymer gels incorporating H-bonding sites such as ureido-triazine, ureido-deazapterin and UPy units are ideal for such purposes. The main properties of these materials were also summarized in recent reviews [100,101].

Complementary H-bonding can also play a key role in adhesion processes as observed between two glass surfaces modified with ureido-7-deazaguanine moieties, using polystyrene films obtained by copolymerization of styrene and a 2,7-diamidonaphthyridine functionalized styrene as the adhesive (see Figure 10 for the H-bonding pattern) [102]. Highly fluorinated linking groups were shown to minimize nonspecific adhesion [103].

### 4.2. Electrochemical and Photochemical Applications

**Figure 27:**
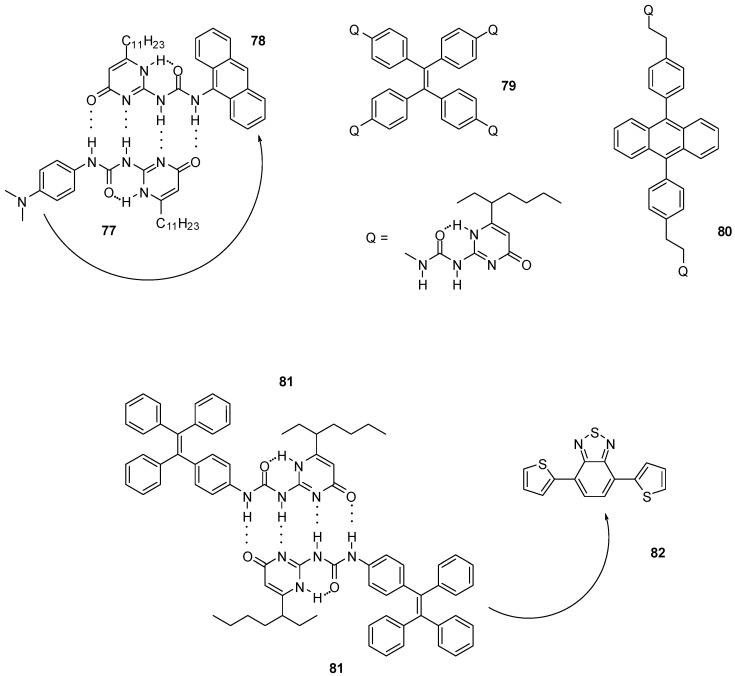
Photo-responsive UPy derivatives **77**–**81**.

UPy derivatives were also applied in the field of photochemistry, photo-physics and electrochemistry. Electronic communication between quadruple H-bonded, electron donating- and accepting monomers (**77**, **78**, Figure 27) can occur via photoinduced electron transfer, which makes them suitable light-energy conversion agents. The attachment of visible light responsive chromophores resulted in a system with fast single-step electron-transfer and slow charge-recombination that could be exploited for light-energy conversion [104].

Polymers constructed of multivalent UPy monomers capable of non-covalent aggregation-induced emission were used for light-harvesting purposes. UPy derivatives of tetra-phenylethylene (**79**, Figure 27) were shown to form supramolecular hyperbranched polymers that could self-assemble further to nanoparticles [105]. The latter were used as energy donors together with sulforhodamine 101 as energy acceptor to construct an artificial light-harvesting system, in which the two components were held together by electrostatic interaction between the dye and positively charged surfactant cetyltrimethylammonium bromide (CTAB) covering the nanoparticles. Similar systems based on dimeric UPy-dibenzyl-anthracene (**80**) [106] or UPy-tetra-phenylethylene units (**81**) [107] were developed that could be assembled to nanoparticles in aqueous media in the presence of surfactants. Upon the addition of different ratios of energy acceptors, e.g., a benzothiadiazole derivative (**82**) in the latter case, tunable emittance could be achieved.

### 4.3. Biomedical Applications

Hydrogels are three-dimensional networks composed of hydrophilic polymer chains that possess the ability to absorb a large amount of water. These materials have widespread biomedical applications [108,109,110]. UPy-containing gels undergo controllable gelation and display self-healing properties due to their great number of repeating complementary binding motifs. Moreover, they show great biocompatibility and thus can be applied even in living organisms, so they can be ideal for use in tissue engineering and drug release. As an example, no macroscopic adverse effects could be detected after intraperitoneal administration of UPy-modified poly(ethylene-glycol) hydrogels in rats [111].

Bioactive materials were obtained by mixing polymer chains with UPy end-groups and UPy-derivatized peptides, such as oligopeptides, with cell adhesion promoting effect. In the in vivo experiments carried out by subcutaneous implantation in rats, vascularization and infiltration of macrophages and, after a couple of days, even the formation of large giant cells were observed [112].

Hyaluronic acid modified with side chains incorporating UPy moieties showed self-healing and some other advantageous properties, such as improved lubrication, enhanced free-radical scavenging, attenuated enzymatic degradation and improved in vivo retention [113].

**Figure 28:**
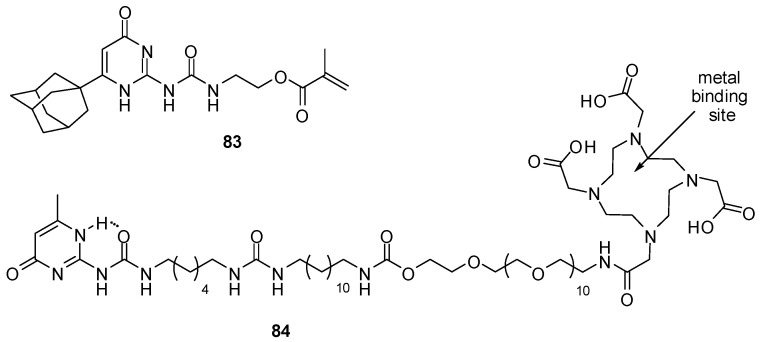
UPy derivatives used to construct hydrogels.

Polymers, obtained by the co-polymerization of **83** (Figure 28) and *N*,*N*-dimethylacrylamide, were shown to form transparent hydrogels that could be injected through a syringe needle without losing their rheological characteristics [114]. The multiple hydrogen bonds could be shielded effectively by the bulky hydrophobic adamantyl moiety. It was proposed that the material could be used as injectable substitute for the eye’s vitreous humor as, after injection in the vitreous cavity, they could recover their gel-like properties. Preliminary in vitro evaluation showed no cytotoxicity.

Another injectable hydrogel was obtained by incorporating UPy units randomly into ABA-type triblock copolymers built from central poly(ethylene-oxide) and terminal poly(methyl-methacrylate) blocks. Drug release from the hydrogel was investigated using bovine serum albumin as a model [115].

Hydrogels formed from poly(ethylene-glycol) chains coupled with UPy end-groups via alkyl-urea spacers could be switched to a viscous liquid at pH > 8.5, which made it possible to pass through a catheter used during intervention to treat myocardial infarction [116]. At the same time, it could form a hydrogel upon contact with the tissue. Also, a growth factor could be incorporated into the hydrogel and its release could be controlled to reduce scar collagen in myocardial infarction in pigs.

Poly(ethylene-glycol) chains end-capped with self-dimerizing ureido-cytosine moieties (see Figure 14) were shown to assemble into nanospheres maintaining structural integrity in a serum-containing biological medium [117]. These structures could be used as drug carriers and induced selective apoptosis in cancer cells in vitro.

UPy-modified gelatin derivatives showed self-healing properties and, according to in vitro studies, they were capable of release of metronidazole, an antimicrobial drug [118], as well as 5-fluorouracil [119]. In the latter case, high UPy content and the presence of Fe^3+^ ions made it possible to control drug release. Rheological investigation of a similar system showed that gel formation was the result of ionic coordination, while strength and self-healing property could be connected to the UPy moieties [120].

In vivo radioactive imaging of hydrogels in a porcine heart was realized by introducing an ^111^In-binding moiety to a hydrogel precursor (**84**, Figure 28) [121].

Hybrid hydrogels, composed of conductive polyaniline chains, thermo-responsive and self-healing UPy-decorated poly(4-styrenesulfonate) chains and Fe^3+^ ions, were found to monitor vibrations created by human vocal chords, pulse beating or finger bending, even after damaging and recovery, so they may have great potential in construction of wearable devices [122]. Due to the easy formation of complementary H-bonds, even infrared irradiation from human skin is sufficient to promote the self-healing of UPy elastomers that can be used in wearable electronics to monitor different motions [123]. A nanocomposite sensor obtained from UPy-decorated multi-walled carbon nanotubes and polymer networks with UPy units, was shown to respond to various deformations, including stretching, bending, and twisting [124].

In order to form modular multi-layered scaffolds for tissue engineering, hierarchical fibrous architectures were constructed from UPy-functionalized polymers and bioactive UPy-peptide conjugates [125] by electrospinning [126,127]. It was shown, that by a modular approach using different UPy-modified building blocks, both cell-adhesive and non-cell adhesive characters could be incorporated into a single bi-layered scaffold.

### 4.4. Applications in Catalysis

**Figure 29:**
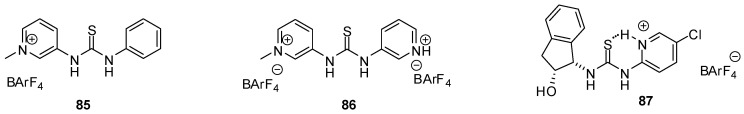
Pyridinium-thiourea derivatives **85**–**87** with enhanced catalytic activity.

Based on their H-bonding properties, urea derivatives are widely used as either basic or Lewis acidic organo-catalysts [128], but there are only a few examples for the application of heterocyclic derivatives. H-donor ability of thiourea catalysts could be increased electrostatically, by the quaternarization of the N-atom of the heterocyclic unit attached to the urea core. Enhanced activity of catalysts **85** and **86** (Figure 29) was observed by Kass in Friedel-Crafts alkylation of trans-β-nitro-styrene with *N*-methyl-indole, Diels-Alder reaction between cyclopentadiene and methyl vinyl ketone and in the aminolysis of styrene oxide with aniline [129]. The favorable effect of stronger binding was proved by the theoretical calculations of Shao and Zou [130].

Another useful strategy for enhancing catalytic activity and enantioselectivity of (thio)urea catalysts was found to construct an internal H-bond by protonation of the heterocyclic moiety [131]. Seidel compared the activity and enantioselectivity of a series of protonated heterocyclic(thio)urea catalysts in the asymmetric addition reactions of indoles to nitroalkenes and found pyridinium derivative **87** the most efficient one with this structure.

**Figure 30:**
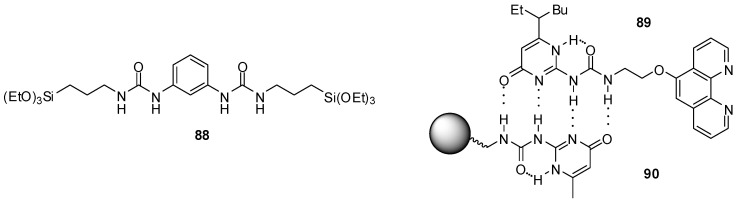
UPy derivatives used to construct recyclable catalysts.

In order to avoid self-aggregation that hinders catalytic effect, the bis-urea derivative **88** (Figure 30) was incorporated into periodic mesoporous organo-silica that prevents dimer formation via isolation of the catalytic units. The co-condensation of **88** with Si(OEt)_4_ in the presence of CTAB led to the formation of mesoporous material that was tested as a heterogeneous catalyst in Henry reactions. In addition to good activity, another advantage of the catalyst is its recyclability [132].

H-bonding ability of UPy derivatives could be exploited even during recycling of a homogenous copper catalyst by introducing a UPy moiety into the ligand (**89**, Figure 30). After the reaction, a domino Sonogashira coupling/cyclization or an azide-alkyne cycloaddition, the solvent was evaporated and a resin with a UPy tag (**90**) was added to the residue of the mixture in CHCl_3_. Thus, due to the formation of H-bonds between the UPy moieties, the catalyst could be filtered. The homogeneous catalyst was cleaved from the resin by washing it with DMF and methanol and, after the evaporation of the solvent, it was reused with only a small loss of activity [133]. Although this methodology requires a rather tedious workup for efficient recycling, it is an interesting example of the utilization of such assemblies.

**Figure 31:**
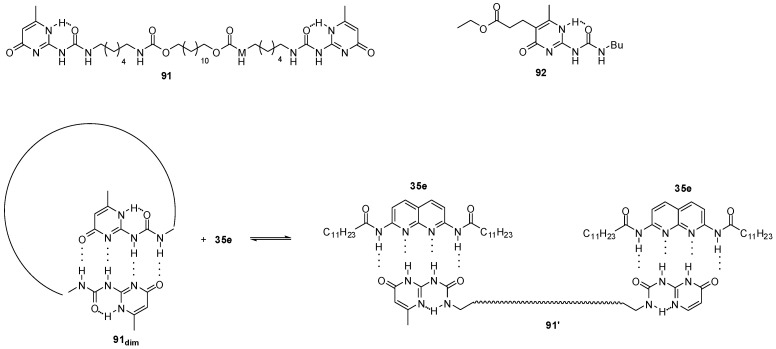
UPy derivatives (**91, 92**) used in the autoregulated catalytic systems for Mannich reactions.

Equilibria between homo- and heterodimerization processes could be used to regulate catalyst concentration in organocatalytic processes in which efficiency and selectivity are usually concentration-dependent. Similarly to the equilibrium depicted in Scheme 5, at high concentrations, a heterodimer structure (**91′**:**35e**_2_, Figure 31) is favored, while upon dilution the homodimer form, a cyclic derivative in the present case (**91_dim_**), is preferred (following Le Chatelier’s principle) and, thus, catalyst **35e** will be released. This means that concentration of the free catalyst will be regulated by the equilibrium between homo- and heterodimeric structures. In the reaction of acetylacetone and trans-β- nitro-styrene, it was proved that the application of UPy derivative **91** and catalyst **35e** in a ratio of 1/2 resulted in an autoregulated system, in which TOF values did not depend significantly on the total concentration of catalyst **35e**, in contrast to the non-regulated variant [134]. Catalytic effect was due to a synergism between naphthyridine **35e** and K_2_CO_3_, so for efficient autoregulation, the concentration of the salt should also be kept constant [135].

Further investigations showed that, while simple UPys had no catalytic effect on Michael additions, the ester functionalized derivative **92** was also able to act as a phase-transfer agent for K_2_CO_3_ (similarly to the naphthyridine derivative **35e**) and, thus, to catalyze Michael additions in CDCl_3_ [136].

### 4.5. Applications in Sensors

Due to their H-bond donor ability, urea and thiourea derivatives are capable of binding various anions, so they can be useful building blocks in sensory devices [137,138]. Thiourea compounds usually form stronger bonds with anionic guests because of the higher acidity of N-H groups. In order to increase the number of interactions between the hosts and guests, the most efficient receptors incorporate multiple urea/thiourea moieties. The introduction of heterocycles can also lead to enhanced binding and/or selective complexation of a specific anion that is often accompanied by structural changes in the urea host.

**Figure 32:**
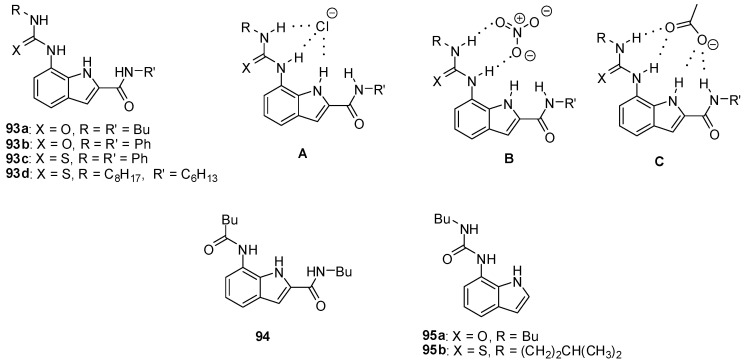
Indolyl receptors (**93**–**95**) and anion complexation with 7-ureido-1*H*-indole-2-carboxamide derivatives (**A**–**C**).

Stronger association can be achieved by the incorporation of heterocycles with NH functionalities to increase the number of H-bonds between hosts and oxo-anion guests, while a lone pair of basic nitrogen atoms may facilitate anion binding by preorganization of the hosts in the proper conformation. The superiority of heterocyclic ureas over other derivatives was shown by the higher affinity of diindolyl-ureas then diphenyl-urea [139], and also by the stronger binding of carboxylates to ureido-indols (e.g., **93a**, Figure 32) than amido-derivatives (e.g., **94**) [140]. Interestingly, on comparing the affinity of urea (**93b**) and thiourea derivatives (**93c**), the more acidic thiourea group showed a lower association constant, probably due to steric hindrance between the large sulfur atom and the aromatic CH groups of the receptor in the conformation necessary for the formation of the proper H-bonding motif.

Based on NMR studies, different complexation of halide- (Figure 32, **A**), nitrate- (Figure 32, **B**) and acetate (Figure 32, **C**) ions by the receptor **93d** could be assumed [141]. The presence of the amido group was essential to bind acetate, but nitrate was found to interact only with the urea functionality. Interestingly, much higher association constants towards halides were obtained for the unsubstituted derivative **95a** (*K*_assoc_(**95a**:Cl^−^) = 4.4 × 10^5^ M^−1^ in CDCl_3_, *K*_assoc_(**93d**:Cl^−^) = 9 × 10^3^ M^−1^ in CDCl_3_). This phenomenon was assumed to be due to steric repulsion and unfavorable dipoles in receptor **93d** after the change from the anti–anti to the syn–syn conformation upon anion binding.

Thiourea derivative **95b** was shown to be a good chloride/bicarbonate exchange agent in phospho-lipide or phospho-lipide/cholesterol membranes. As mis-regulation of either chloride- or bicarbonate transport can result in a number of serious diseases, similar compounds may find applications in medicinal chemistry [142].

**Figure 33:**
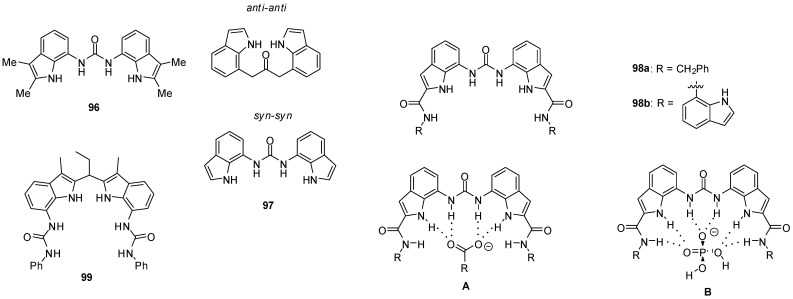
Di-indolyl-urea derivatives (**96**–**99**) used as anion receptors and anion complexation of 2-amido-derivative **98a** (**A**,**B**).

1,3-Diindolylureas (e.g., **96**, Figure 33) were shown to bind oxo-anions, such as acetate, benzoate and dihydrogen phosphate, with *K*_assoc_ > 10^4^ M^−1^ in DMSO-d_6_ containing 0.5% water, but their affinity for chloride was low (*K*_assoc_ = 128 M^−1^). On the increase in the water content to 10%, a considerable selectivity was observed for dihydrogen phosphate (*K*_assoc_(H_2_PO_4_^−^)/*K*_assoc_(AcO^−^) = 8.5). Solid state structures were found to be considerably different from those in solution. X-ray measurements showed that two or three ureas could bind to each anion so the complexes were stabilized by eight or twelve hydrogen bonds, while solution-phase NMR studies proved the formation of 1:1 complexes in most cases. Thiourea receptors were less effective again [140]. It should be mentioned, however, that 1,3-diindolylureas (e. g. **97**) were also capable to bind fluoride ions. According to DFT calculations, the presence of fluoride induced a conformational change from the anti–anti to the syn–syn conformer [143].

Based on the results of NMR titrations, different modes for binding were assumed for complexes of the anion receptor containing six hydrogen bond donor groups (**98a**) with carboxylates (**A**) and dihydrogen phosphate (**B**). Upon addition of dihydrogen phosphate in excess, a proton transfer process may also occur, and the deprotonation of the complexed anion results in the formation of a mono-hydrogen phosphate-receptor complex and H_3_PO_4_ [144].

Strong binding of sulfate could be observed for bis-amide **98b** with *K*_assoc_ > 10^4^ M^−1^ in DMSO-d_6_ containing 10% water, and X-ray structure proved the formation of eight H-bonds with the involvement of urea, indole and amide NH protons [145].

7,7′-Diureido-2,2′-diindolylmethanes showed preferential binding of tetrahedral oxo-anions, such as hydrogen sulfate and dihydrogen phosphate, over chloride or benzoate, with association constants between 235–535 M^−1^ for compound **99** even in methanol [146].

**Figure 34:**
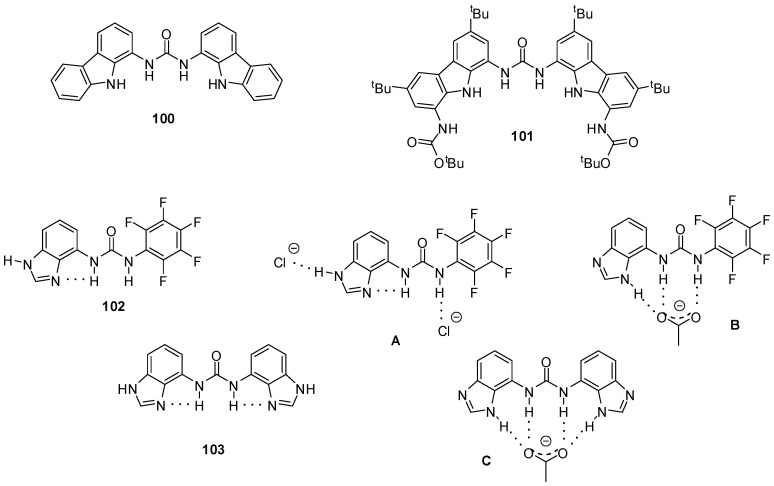
Anion receptors with carbazole (**100**, **101**) and benzimidazole functionalities (**102**, **103**) and anion complexation of benzimidazolyl derivatives (**A**–**C**).

Carbazolyl-ureas were found to be even better receptors for oxo-anions with association constants exceeding 10^4^ M^−1^ for acetate, 5 × 10^3^ M^−1^ for benzoate and 6 × 10^3^ M^−1^ for dihydrogen phosphate (in DMSO-d_6_ containing 0.5% water). Moreover, the fluorescence of compound **100** (Figure 34) could be quenched by benzoate, which makes it an especially useful sensor for this anion [147]. Analogous thiourea derivatives showed weaker binding and lower selectivity [148].

Among other receptors, association constants of diindolyl- and dicarbazolyl ureas for 11 different carboxylate anions were determined [149]. These derivatives were proved to be better acceptors than bis-or tris-ureas lacking heterocyclic functionalities. It was revealed that, beside differences in acidity/basicity of hosts and guests, steric effects had a considerable effect on binding strength. As an example, receptor **100** established only weak interaction with acetate, while it was the best binder for pivalate. This was explained by the perfect fitting of the latter into the cavity of the receptor that allowed for an interaction between the ^t^Bu groups of the anion and the carbazole rings. At the same time, acetate is too small for such an effect. In contrast, carbazole **101** showed similar affinity for both carboxylates: in this case acetate had the right size, while pivalate was too large.

Involvement of different tautomer forms of benzimidazolyl-ureas were observed in complexation with different anions. In the absence of guests, compounds **102** and **103** were stabilized by intramolecular H-bonds that were retained in the presence of chloride ions (Figure 34, **A**). At the same time, the addition of acetate resulted in the formation of the other tautomer that made it possible to develop more interactions between hosts and guests (Figure 34, **B**, **C**) [150].

**Figure 35:**
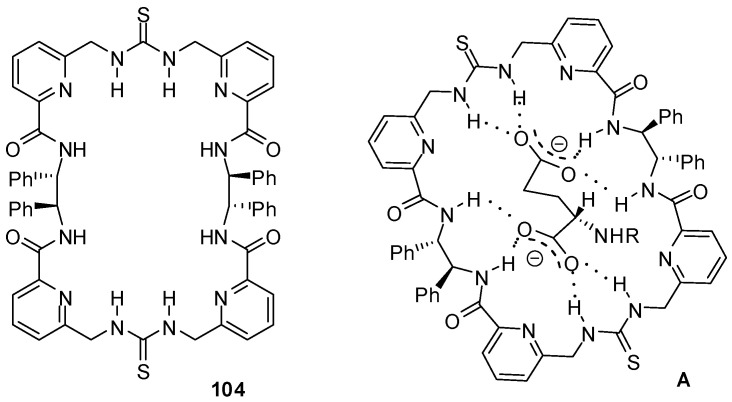
Chiral macrocycle **104** and its complex with L-glutamate (**A**).

The chiral macrocycle **104** (Figure 35) showed a preference to bind *N*-protected L-aspartate and L-glutamate over the D-enantiomers with *K*_assoc_ > 10^4^ M^−1^ in CH_3_CN or DMSO [151]. According to DFT studies, eight H-bonds could be established between the host and the L-amino acid derivatives.

**Figure 36:**
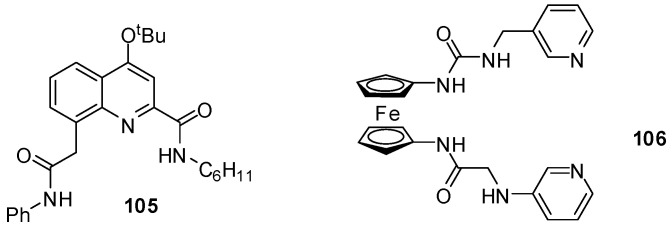
Fluoride sensors.

Quinoline derivatives with various substituents in the amide and urea functionalities were found to be excellent and selective receptors for fluoride ions, with *K*_assoc_ = 1.5 × 10^5^ M^−1^ (in CDCl_3_) for **105** (Figure 36) [152]. The difference in the binding strength for different anions was explained by the size of the cavity that accommodated well the small fluoride anion but not the larger anions that had to be located above the plane of the receptor.

Behavior of the ferrocene-containing heteroditopic receptor **106** towards a series of anions could be investigated not only by NMR titrations, but also by UV–Vis spectroscopy and CV. Cathodic shift was observed only in the presence of fluoride ions and, to a lesser extent, upon addition of H_2_PO_4_^−^. Also, a salient association constant (*K*_assoc_ = 9 × 10^6^ M^−1^ in DMSO-d_6_) was measured during complexation of the former anion [153].

**Figure 37:**
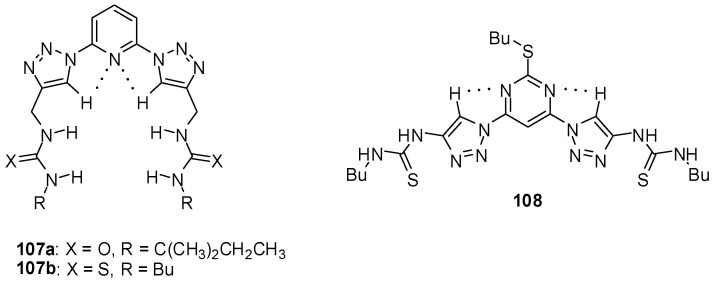
Triazolyl receptors for chloride and bromide ions.

During the investigation of a series of triazolyl receptors, ureido derivatives were shown to have higher affinity towards chloride and bromide ions than the analogous amide type compounds [154]. The incorporation of the pyridyl group resulted in a preorganization of the hosts **107a,b** (Figure 37) that led to increased interaction than that observed for the pyrimidine-type compound (**108**) adopting another conformation in the non-complexed form. Thiourea receptors had slightly higher association constants than urea derivatives (*K*_assoc_(**107a**:Cl^−^) = 1.0 × 10^3^ M^−1^, *K*_assoc_(**107b**:Cl^−^) = 2.5 × 10^3^ M^−1^, *K*_assoc_(**108**:Cl^−^) = 1.7 × 10^2^ M^−1^ in CD_3_CN).

As another advantage of the heterocyclic moieties, they may ensure the binding of cations, which, together with the urea functionality, results in a complex formation with anion-cation pairs.

**Scheme 16:**
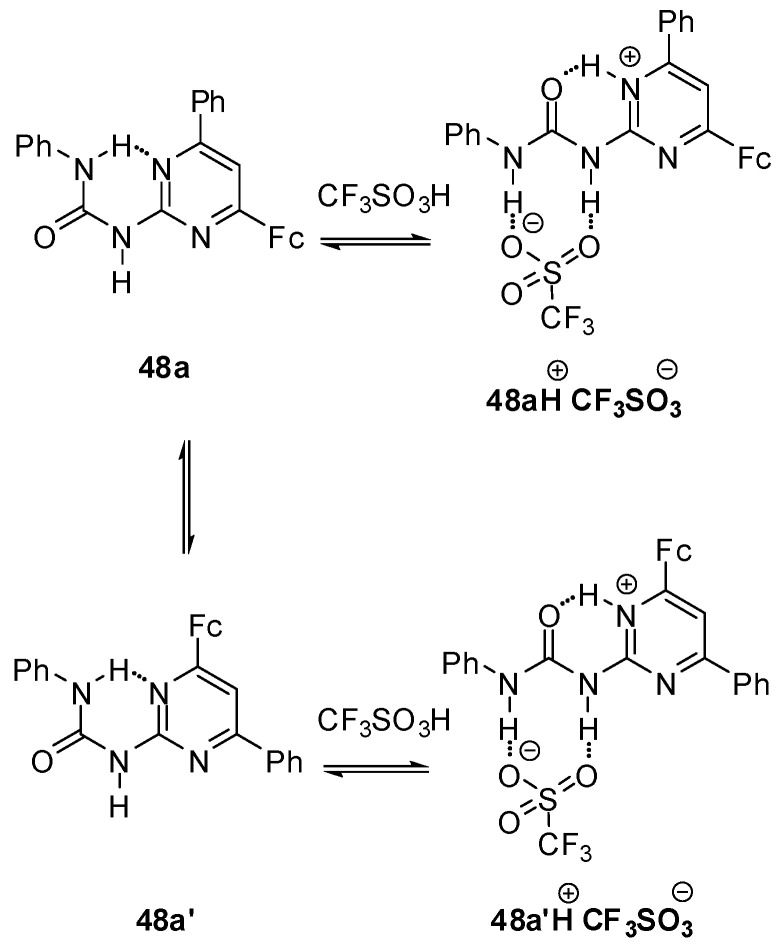
Acid-induced conformation change in ferrocenyl derivative **48**.

Reversible protonation could be followed by a change in the color of solution of ferrocenyl ureido-pyrimidine **48a** from orange to violet and back upon multiple protonation/deprotonation cycles [155]. The addition of acids resulted in a conformational change in the ferrocene derivative leading to a DDA H-bonding array (**48aH^+^**, Scheme 16) that can serve as a proper binding site for the anion of the acid. In contrast to the non-protonated form, the slow exchange between the two conformers (**48aH^+^** and **48a′H^+^**) of the complexes obtained with CF_3_SO_3_^−^ and BF_4_^−^ anions made them distinguishable even at room temperature. At the same time, separate signals of the two isomers could be detected only by low temperature ^1^H NMR experiments in the presence of CF_3_CO_2_H, which showed a rapid equilibrium between non-protonated, complexed and protonated (decomplexed) forms. The differences were also reflected in the electrochemical behavior of the complexes. Gradually decreasing levels of peak currents and a new peak at higher potential with increasing intensity were observed in the CVs obtained by the titration of **48a** either with CF_3_SO_3_H or HBF_4_. In contrast, a gradual shift of both the cathodic and anodic peak could be measured in the presence of increasing amounts of CF_3_CO_2_H.

**Figure 38:**
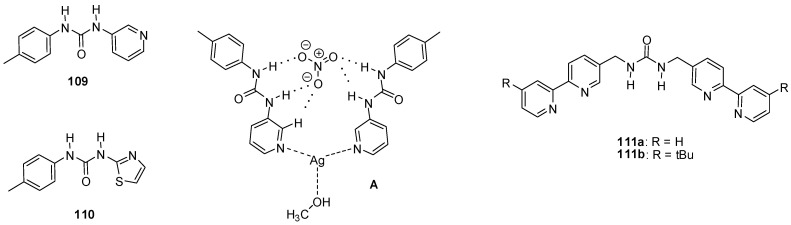
Urea derivatives used to bind metal salts (**109**–**111**) and the structure of the [Ag(**109**)_2_](NO_3_) · MeOH assembly (**A**).

The formation of an [Ag(**109**)_2_](NO_3_)∙MeOH assembly (Figure 38**A**) was observed upon addition of AgNO_3_ to urea **109** in a methanol/water = 1/1 mixture, while CF_3_SO_3_^−^ and SO_4_^2−^ salts did not form analogous species [156]. This complexation might find application in ion sequestration as was shown for Cu(NO_3_)_2_ using a polymer bound variant of **109** as the trapping agent [157].

The [Ag(**110**)_2_]^+^ host, obtained from an analogous thiazolyl derivative, could form cage-like host-guest complexes with 2:1 stoichiometry with SO_4_^2−^ and SiF_6_^2−^ ions even in DMSO, a highly competitive medium [158].

Metal-organic cages with M_4_L_6_ (M = Ni, Zn) stoichiometry, obtained via coordination-driven self-assembly, were shown to be capable of binding tetrahedral EO_4_^n−^ type oxo-anions (E = S, Se, P, Cr, Mo, W) [159,160]. In these tetrahedral cages, the four metal ions were in the vertices and the ligands occupied the edges of the tetrahedron. The bipyridyl units of the ligands served as coordination sites for the metal ions and the urea functionalities were responsible for anion binding in the cavity. The self-assembly was found to be templated by the anions, and upon removal of the latter the cages rearranged into different assemblies. Due to this instability in the absence of anions, only a lower limit could be estimated for the association constant of the Ni_4_(**111a**)_6_^8+^ cage for sulfate binding (*K*_assoc_> 6 × 10^6^ M^−1^). In case of the [Zn_4_(**111b**)_6_(EO_4_)]^6+^ cages, relative anion encapsulation selectivity followed the order of PO_4_^3−^ ≫ CrO_4_^2−^ > SO_4_^2−^ > SeO4_2−_ > MoO_4_^2−^ > WO_4_^2−^ as determined by NMR experiments.

**Figure 39:**
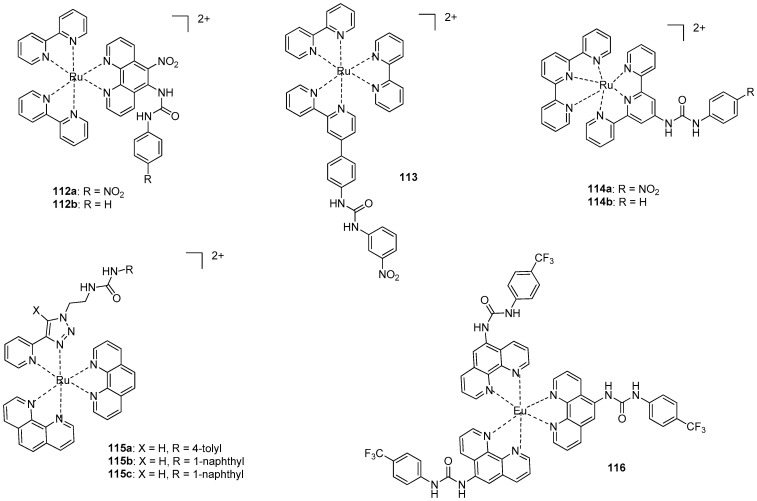
Ru-and Eu-based colorimetric sensors for anions.

Coordination of metals to the heterocyclic functionality may also make it possible to follow anion binding by photochemical methods. In this regard, the applicability of several Ru-complexes was tested. Emission spectral studies showed that the emission intensity of complex **112a** (Figure 39) was completely quenched (‘switched off’) in the presence of an excess of F^−^, CH_3_COO^−^ and H_2_PO_4_^−^ ions, but only a small change in the intensity of emission was observed for Br^−^, Cl^−^, HSO_4_^−^ or I^−^ [161]. H-bonded adducts with 1:1 stoichiometry were formed at relatively low concentration of the first three anions, while at higher concentrations a deprotonation of the receptor took place. The same conclusions could be drawn from experiments carried out with the Ru-complex **112b**, but association constants were lower [162] (e.g., *K*_assoc_(**112a**:F^−^) = 2.2 × 10^5^ M^−1^. *K*_assoc_(**112b**:F^−^) = 6.5 × 10^4^ M^−1^ in CH_3_CN).

In contrast to the previous examples, no change was observed in the emission of complex **113** in the presence of fluoride [163]. Phosphate anions could be detected selectively over other anions, such as acetate and sulfate. Moreover, the receptor could differentiate between phosphate and pyrophosphate, as the former caused an increased emission, and, on the contrary, emission could be quenched in the presence of the latter.

Ru-terpyridine complexes **114a,b** showed an affinity order of different anions of Cl^−^ > NO_2_^−^ > Br^−^ > NO_3_^−^ > I^−^ [164]. Similarly to the previous observations, binding constants for the nitro derivative **114a** were almost an order of magnitude larger (*K*_assoc_(**114b**:Cl^−^) = 4.5 × 10^5^ M^−1^. *K*_assoc_(**114a**:Cl^−^) = 2.0 × 10^6^ M^−1^, *K*_assoc_(**114b**:I^−^) = 9.5 × 10^3^ M^−1^. *K*_assoc_(**114a**:I^−^) = 2.5 × 10^4^ M^−1^ in CH_3_CN).

The Ru-complex with the anion binding triazolyl-urea unit (**115a**) could form complexes with phosphates and carboxylates [165]. It was proved by NMR studies that the strongly basic phosphate ions were bound by both urea NH and triazol CH groups, while carboxylates formed H-bonds only with the urea moiety. Phosphate anions could selectively be sensed in the presence of other competitive anions via enhancement of the emission of the complex. Similar behavior was observed with complexes **115b** and **115c**, but association constants with the latter were an order of magnitude larger [166].

The addition of AcO^−^, H_2_PO_4_^−^, Cl^−^, Br^−^ and I^−^, ions into the solution of Eu-complex **116** led to significant changes in the Eu(III) emission [167]. Moreover, it was fully quenched, in the presence of AcO^−^ and H_2_PO_4_^−^. Titration with F^−^ resulted in an initial enhancement of emission up to one equivalent, followed by quenching when the anion was in excess. This was explained by initial binding of the anion to the metal center that led to an increase in the Eu(III)emission, followed by quenching of the emission by complex formation between the anion and the urea moieties.

**Scheme 17:**
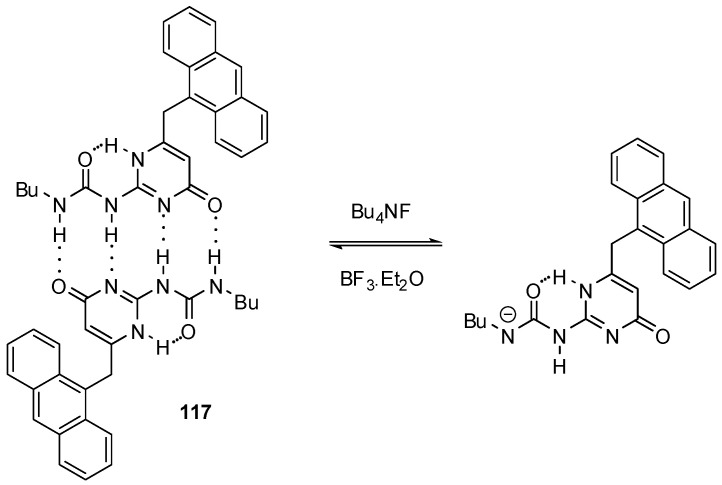
Deprotonation of UPy derivatives **117** in the presence of fluoride ions.

In contrast to the applications discussed above, self-assembly of molecules with a UPy moiety was utilized during the construction of a fluoride sensor **117** (Scheme 17) [168]. The 9-methylene-anthracene unit made it possible to determine binding strength by fluorescence titration. Addition of less than one equivalent of fluoride led to a small decrease in the intensity of fluorescence, while the presence of the anion in excess led to quenching, i.e., the formation of a non-emissive complex. This phenomenon was explained by the deprotonation of the UPy derivative **117** and formation of the very stable HF_2_^−^ anion. Other halide ions induced no changes in the spectra.

## 5. Summary and Outlook

The most important property of urea-functionalized heterocycles is their capability to establish multiple H-bonds to form either homodimers or host–guest complexes with other molecules bearing a complementary H-bonding array. The bonding pattern is often influenced by conformational changes and the existence of different tautomeric forms. At the same time, with the choice of a suitable heterocycle as well as the introduction of other functionalities, the structure can often be pushed towards the array necessary for a given interaction. Although strongest binding can mostly be achieved by UPys, ureido derivatives of other heterocycles, bearing triple- or quadruple H-bonding motifs, can also be useful elements in different assemblies. Moreover, the heterocycles may perform a number of other functions beyond offering further H-bond donor/acceptor groups: they can be used as metal binding sites, redox responsive or photo-switchable elements. Bifunctional molecules can serve as monomers in supramolecular polymers, often with self-healing properties. The advantages of such systems in material science applications have been proved. In addition, their biocompatibility and advantageous mechanical properties make them ideal candidates for biomedical applications from drug release to tissue engineering. This is among the most rapidly expanding fields of research concerning ureido-heterocycles. There are some really promising results and the utility of some methodologies was proved even in in vivo experiments. At the same time, it there is still a long way to go to achieve applicability in human medicine.

Another interesting area is the application of urea-functionalized heterocycles as building blocks in sensory devices where redox- pH- and photoactivity of the heterocycles can be prominently profitable. Complexation of anions, mainly that of oxo-anions, is a well-explored field; at the same time, sensing of neutral molecules, especially in aqueous media, is more problematic. In this regard, the introduction of the proper functionalities to ensure the formation of hydrophobic pockets, as is shown by some examples for aggregation in water, seems to be essential.
